# Targeting the PHB2–ACSL3 Lipid‐Remodeling Axis Overcomes Cisplatin Resistance by Restoring Ferroptosis in Gastric Cancer

**DOI:** 10.1002/advs.77350

**Published:** 2026-08-21

**Authors:** Liang Xu, Wanying Xiang, Xinyue Wang, Yue Pan, Jing Gao, Jiezhen Yang, Yufei Wang, Zhipu Zhu, Man Tong, Lei Jin, Yan Ye

**Affiliations:** ^1^ Department of Immunology School of Basic Medical Sciences Anhui Medical University Hefei China; ^2^ State Key Laboratory of Metabolic Dysregulation & Prevention and Treatment of Esophageal Cancer Tianjian Laboratory of Advanced Biomedical Sciences School of Convergence Medicine Zhengzhou University Zhengzhou China; ^3^ School of Biomedical Sciences The Chinese University of Hong Kong Hong Kong China; ^4^ Huai’an Central Blood Station Huai’an China; ^5^ Department of Pathology Zhongshan Hospital (Xiamen Branch) Fudan University Xiamen China; ^6^ Department of Pathology The Second Affiliated Hospital of Anhui Medical University Hefei China

**Keywords:** ACSL3, ferroptosis, gastric cancer, PHB2, phospholipid remodeling

## Abstract

Platinum‐based chemotherapy resistance remains a major obstacle in gastric cancer (GC) treatment. Through integrated transcriptomic profiling of cisplatin‐resistant xenografts and pharmacogenomic interrogation of the NCI‐60 dataset, we identified Prohibitin‐2 (PHB2) as a previously unrecognized determinant of chemoresistance. PHB2 was consistently upregulated in resistant tumors and associated with poor clinical outcomes. Mechanistically, PHB2 binds the lipid‐metabolic enzyme ACSL3 through a defined AMP‐binding–domain interface (residues W244/H254/E260), enhancing ACSL3 activity to promote monounsaturated fatty acid incorporation into phospholipids. This phospholipid remodeling suppresses lipid peroxidation and establishes a ferroptosis‐resistant state. Structure‐based virtual screening of an FDA‐approved drug library nominated the CXCR4 antagonist Mavorixafor as a potent inhibitor of the PHB2–ACSL3 interaction. Mavorixafor disrupted lipid remodeling, restored ferroptosis susceptibility, and resensitized cisplatin‐resistant GC in cell line–derived xenografts (CDOs) and patient‐derived organoids (PDOs). The therapeutic effect was abrogated by the ferroptosis inhibitor Liproxstatin‐1, confirming ferroptosis dependence. Collectively, our findings define a PHB2–ACSL3 lipid‐metabolic axis that drives ferroptosis escape and identify Mavorixafor repurposing as an immediately translatable strategy to overcome cisplatin resistance in treatment‐refractory GC.

## Graphical abstract

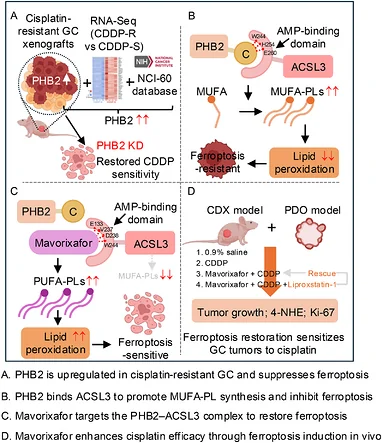

## 1 Introduction

Platinum‐based chemotherapy remains a cornerstone of treatment for gastric cancer (GC), a disease that continues to impose a major global health burden [1, 2]. However, intrinsic or acquired resistance to cisplatin and related agents frequently arises, severely limiting long‐term therapeutic efficacy and contributing to poor patient outcomes [3]. Extensive efforts have been made to elucidate the molecular mechanisms underlying chemoresistance, with emphasis on enhanced DNA repair capacity, upregulated drug efflux, and evasion of apoptosis [3, 4, 5]. Yet, these canonical pathways do not fully account for the marked heterogeneity and persistence of resistance observed in clinical tumors. This has prompted investigation into alternative, non‐apoptotic cell death mechanisms that may be co‐opted during cancer progression and treatment failure.

Among these, ferroptosis, a regulated form of necrotic cell death driven by iron‐dependent lipid peroxidation, has garnered increasing attention as a critical determinant of therapy response across multiple cancer types [6, 7, 8]. Unlike apoptosis, ferroptosis is characterized by the accumulation of reactive oxygen species (ROS) and the oxidative destruction of membrane phospholipids enriched in polyunsaturated fatty acids (PUFAs), ultimately resulting in membrane disruption and cell death [9, 10]. Recent studies have implicated ferroptosis suppression as a hallmark of drug‐tolerant tumor states and a key barrier to effective chemotherapy [8, 11].

Ferroptotic sensitivity is closely governed by the composition and remodeling of membrane lipids [12]. Phospholipids containing PUFAs are highly susceptible to peroxidation and promote ferroptosis, whereas incorporation of monounsaturated fatty acids (MUFAs) into phospholipids reduces susceptibility to lipid peroxidation and confers resistance [10, 12]. This remodeling is orchestrated by the acyl‐CoA synthetase long‐chain family, particularly ACSL4, which activates PUFAs and sensitizes cells to ferroptosis, and ACSL3, which activates MUFAs to suppress ferroptotic signaling [13, 14, 15, 16]. While the role of ACSL4 in promoting ferroptosis has been well characterized, the upstream regulation and biological relevance of ACSL3 remain largely unexplored [14, 17].

To address this gap, we established a cisplatin‐resistant (CDDP‐R) GC xenograft model and performed transcriptomic profiling of resistant tumors. By integrating these data with drug sensitivity profiles from the NCI‐60 CellMiner pharmacogenomic database, we identified a subset of genes whose expression inversely correlated with cisplatin responsiveness. Among these, Prohibitin 2 (PHB2) emerged as a leading candidate. PHB2 is a highly conserved scaffold protein traditionally associated with mitochondrial architecture, nuclear transcriptional regulation, and stress adaptation [18, 19, 20, 21, 22]. However, recent studies suggest that PHB2 can also interact with enzymes involved in lipid metabolism and regulate cytoplasmic signaling events. These features are consistent with a moonlighting role beyond its canonical mitochondrial functions [18, 19, 23, 24, 25]. Whether this noncanonical activity contributes to therapy resistance through metabolic remodeling remains to be determined.

Here, we uncover a previously unrecognized role for PHB2 in mediating ferroptosis resistance through direct activation of the lipid‐metabolic enzyme ACSL3. By enhancing MUFA incorporation into membrane phospholipids, PHB2 suppresses lipid peroxidation and promotes chemotherapy resistance in GC. Through structure‐based virtual screening of a chemical library, we identify Mavorixafor, an FDA‐approved CXCR4 antagonist [26], as a potent disruptor of the PHB2‐ACSL3 interaction. Mavorixafor reprograms lipid composition, restores ferroptotic sensitivity, and overcomes cisplatin resistance in both xenograft and patient‐derived organoid models.

These findings reveal a previously unrecognized lipid remodeling axis driven by a moonlighting function of PHB2 and highlight drug repurposing of Mavorixafor as a clinically actionable strategy to overcome ferroptosis resistance in treatment‐refractory GC.

## 2 Results

### 2.1 Upregulation of PHB2 Drives Cisplatin Resistance in GC

To identify molecular drivers of chemotherapy resistance in GC, we generated a clinically relevant cisplatin‐resistant (CDDP‐R) AGS xenograft model by engrafting in vitro‐selected CDDP‐R cells into mice (Figure S1A,B). Compared with cisplatin‐sensitive (CDDP‐S) counterparts, CDDP‐R xenografts displayed pronounced resistance to CDDP (Figure S1C–E), a frontline platinum‐based chemotherapeutic widely used in GC treatment [2, 27, 28], without evidence of systemic toxicity, as indicated by stable body weight across treatment groups (Figure S1F).

**FIGURE 1:**
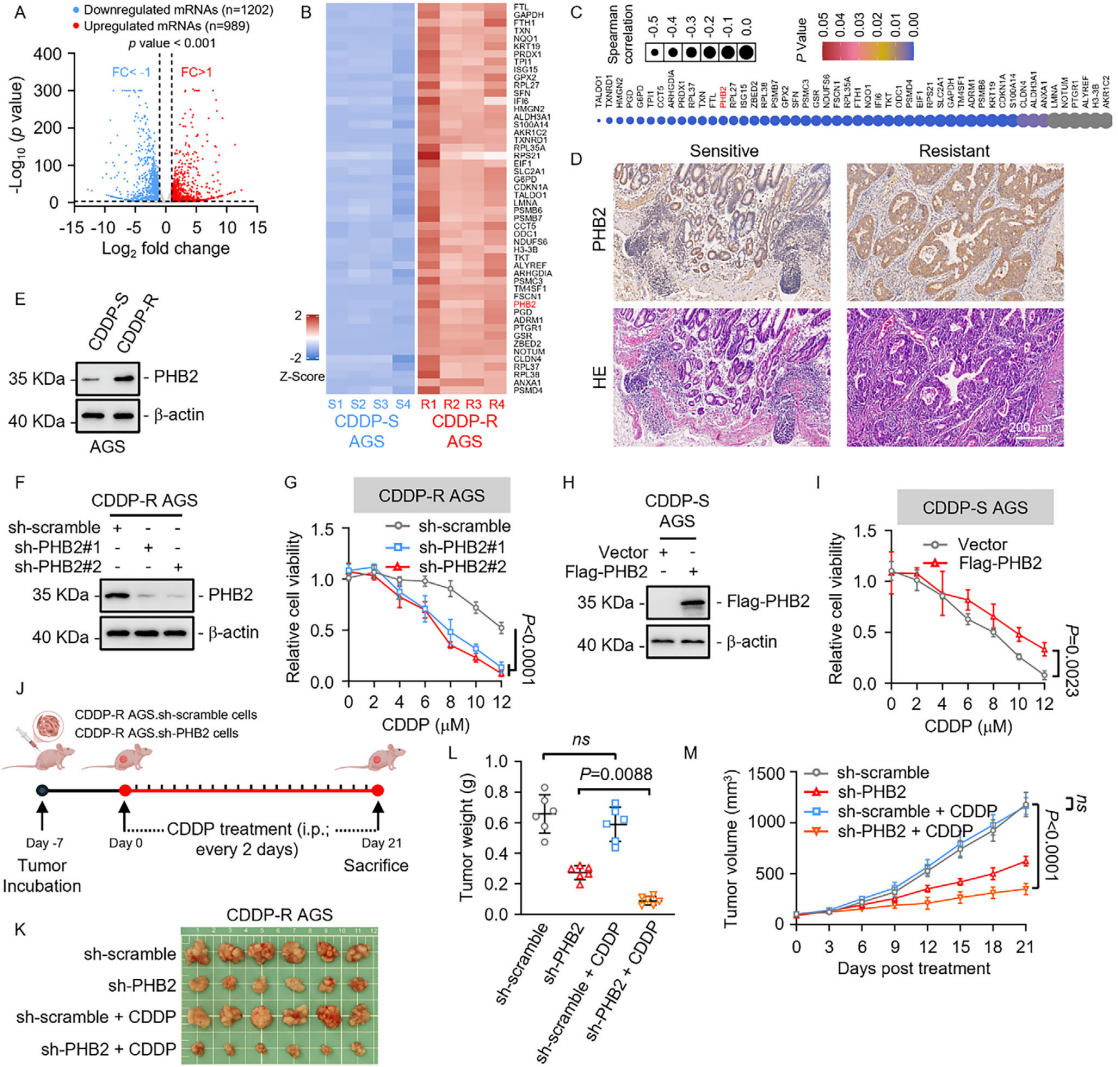
High PHB2 expression promotes chemotherapy resistance in GC. A,B) Volcano plot (A) of differentially expressed proteins and heatmap (B) of the top 50 proteins in CDDP‐R versus CDDP‐S AGS xenograft tumors (fold change >1 or <–1, *P* < 0.001; n = 4 mice per group). C) Dot plot showing the correlation between expression of selected genes and CDDP sensitivity (GI50 values) across the NCI‐60 cell line panel, based on CellMiner analysis. Each dot represents a gene, with size indicating the Spearman correlation coefficient and color corresponding to the statistical significance (*P* value). Genes are ranked by correlation strength. PHB2 is highlighted in red, exhibiting a strong negative correlation with CDDP sensitivity. D) IHC staining analysis of PHB2 expression in GC tissues from cisplatin‐sensitive (CDDP‐S, n = 10) and cisplatin‐resistant (CDDP‐R, n = 24) patient groups. Representative images are shown. Scale bar, 200 µm. E) Western blot analysis of PHB2 expression in CDDP‐S and CDDP‐R AGS cells. β‐actin was used as control. F,G) Western blot examined PHB2 knockdown efficiency in CDDP‐R AGS cells transfected with sh‐PHB2 (F), and subsequent MTT assay showed reduced cell viability after 72 hours of CDDP treatment (G). H,I) Overexpression of PHB2 (H) enhanced resistance to CDDP‐induced cytotoxicity, as assessed by MTT assay after 72 hours of CDDP treatment (I). J–M) Schematic illustration of the establishment of CDDP‐R AGS.sh‐scramble and AGS.sh‐PHB2 xenograft tumors. BALB/c nude mice bearing CDDP‐R AGS‐derived xenografts were treated with either 0.9% saline or CDDP (5 mg/kg, intraperitoneally [i.p.]) every 2 days for 3 weeks. n  =  6 mice per group (J). Representative images (K), tumor weight (L), and growth curves (M) of AGS.sh‐scramble and AGS.sh‐PHB2 xenografts in BALB/c nude mice. Scale bar, 1 cm. Data represent three independent experiments and are presented as mean ± SD. Quantitative data were normalized to the corresponding sh‐scramble control (G,I). One‐way (L) or two‐way ANOVA followed by Tukey's multiple comparison (G, I, M).

RNA‐sequencing (RNA‐seq) comparing CDDP‐R and CDDP‐S tumors revealed 989 transcripts significantly upregulated and 1,202 downregulated in resistant tumors (Figure 1A). Heatmap analysis of the top 50 upregulated genes revealed a distinct expression signature in resistant tumors (Figure 1B). To link these transcriptional alterations to drug response, we integrated the differentially expressed genes with gene‐drug sensitivity correlation profiles from the NCI‐60 CellMiner database (https://discover.nci.nih.gov/cellminer/). This analysis highlighted several candidates whose expression inversely correlated with CDDP sensitivity (Figure 1C), among which Prohibitin 2 (PHB2) emerged as a top hit: its mRNA levels were markedly elevated in CDDP‐R xenografts and negatively correlated with CDDP response across multiple cancer cell lines.

Immunohistochemistry (IHC) of GC patient specimens showed significantly higher PHB2 protein expression in tumors from CDDP‐R patients compared with CDDP‐S cases (Figure 1D). Western blotting confirmed elevated PHB2 protein in CDDP‐R AGS cells (Figure 1E). Notably, our previous work demonstrated that high PHB2 expression correlates with poor overall survival in GC patients [24], further underscoring its clinical relevance.

To functionally evaluate its role in chemotherapy resistance, we silenced PHB2 using two independent shRNAs in CDDP‐R AGS cells (Figure 1F). PHB2 knockdown markedly increased cisplatin sensitivity, as evidenced by reduced cell viability (Figure 1G) and impaired clonogenic growth (Figure S1G). Conversely, ectopic PHB2 expression in CDDP‐S cells (Figure 1H) enhanced resistance, promoting cell survival and colony formation following treatment (Figure 1I; Figure S1H).

To further evaluate the in vivo relevance of PHB2 in promoting chemoresistance, CDDP‐R cells stably expressing sh‐PHB2 or sh‐scramble were subcutaneously implanted into BALB/c nude mice, followed by saline or cisplatin treatment (Figure 1J). Mice bearing PHB2‐depleted tumors exhibited reduced tumor volume and weight in response to cisplatin (Figure 1K–M).

### 2.2 Ferroptosis Suppression by PHB2 Underlies Cisplatin Resistance in GC

**FIGURE 2:**
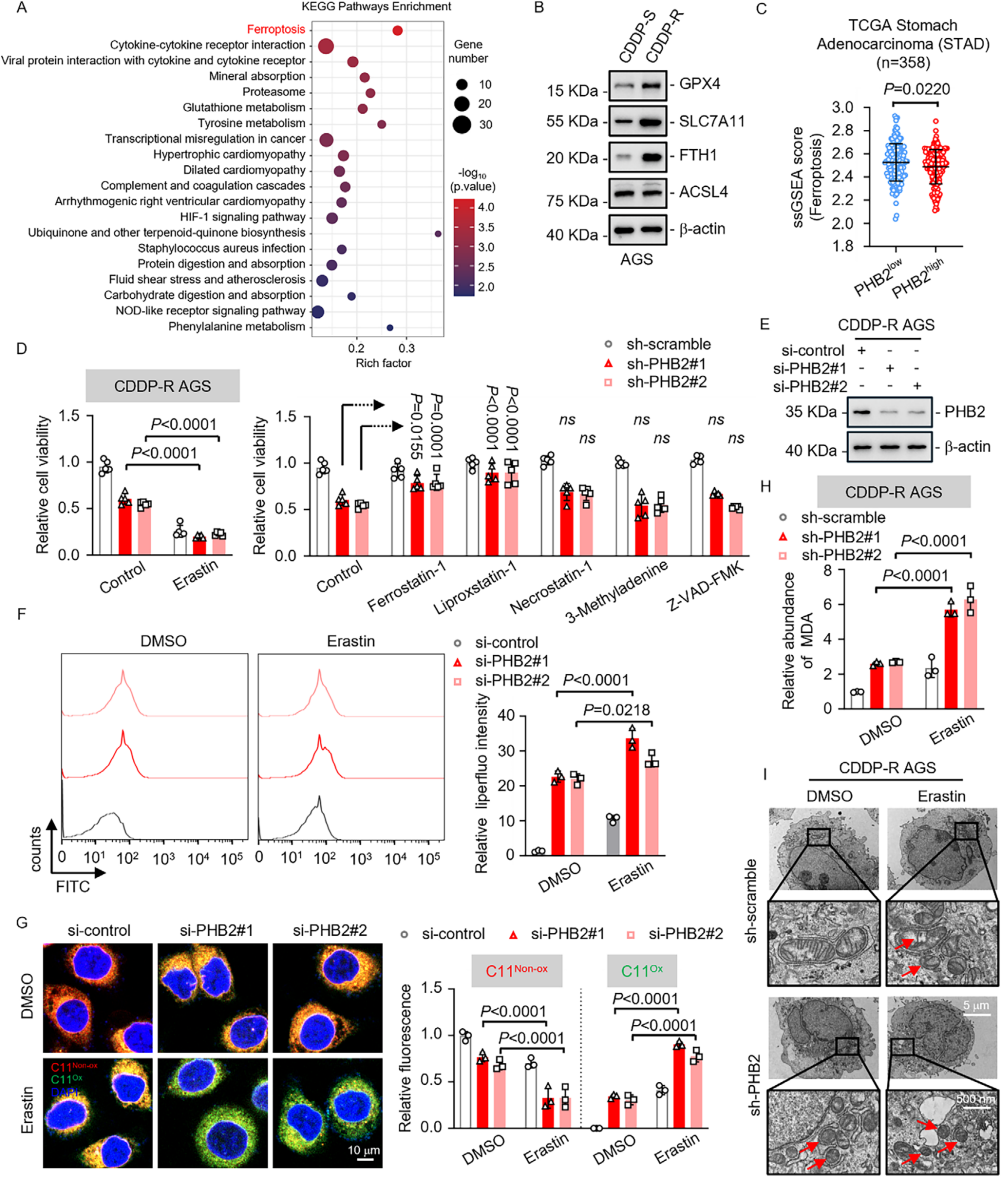
PHB2 drives chemotherapy resistance in GC by regulating ferroptosis. A) KEGG pathway enrichment analysis revealed that ferroptosis was significantly enriched in CDDP‐S compared to CDDP‐R AGS xenograft tumors. Dot size represents the number of genes in each KEGG pathway; KEGG, Kyoto Encyclopedia of Genes and Genomes. B) Western blot analysis of ferroptosis‐related genes GPX4, SLC7A11, FTH1, and ACSL4 in CDDP‐S and CDDP‐R AGS cells, with β‐actin used as control. C) ssGSEA scores for the ferroptosis pathway in low and high PHB2 expression clusters in TCGA Stomach Adenocarcinoma (STAD) cohort (n = 358). D) CDDP‐R AGS cells were treated for 72 hours with pathway‐specific compounds, including the ferroptosis inducer Erastin; ferroptosis inhibitors Ferrostatin‐1 and Liproxstatin‐1; the necroptosis inhibitor Necrostatin‐1; the autophagy inhibitor 3‐Methyladenine; and the apoptosis inhibitor Z‐VAD‐FMK. Cell viability was then evaluated by MTT assay. E–G) Western blot analysis confirmed PHB2 knockdown efficiency in CDDP‐R AGS cells transfected with si‐PHB2 (E). Lipid peroxidation, assessed using Liperfluo (F) and BODIPY 581/591 C11 (G) staining, was elevated following PHB2 knockdown, especially in the Erastin‐treated group. H) MDA level analysis revealed a significant increase upon PHB2 knockdown, which was further enhanced by Erastin treatment. I) TEM images showing mitochondrial morphology and cristae structure in Erastin‐treated CDDP‐R AGS.sh‐scramble and AGS.sh‐PHB2 cells. Scale bar, 5 µm (top) and 500 nm (bottom). Data represent three independent experiments and are presented as mean ± SD. Quantitative data were normalized to the corresponding control (D,F–H). Two‐tailed Student's *t*‐test (C). One‐way ANOVA followed by Tukey's multiple comparison (D,F–H).

To elucidate the mechanism by which PHB2 promotes chemotherapy resistance in GC, we performed KEGG pathway enrichment analysis on RNA‐seq data from CDDP‐S and CDDP‐R AGS xenograft tumors. Remarkably, ferroptosis emerged as the most significantly enriched pathway (Figure 2A), consistent with its emerging role in therapy resistance across multiple cancers [7, 8]. We next examined the expression of key ferroptosis‐related genes [29, 30, 31], including *GPX4*, *SLC7A11*, *FTH1*, and *ACSL4*, in the RNA‐seq dataset (Figure S2A–D), and assessed their protein levels in xenograft tumor lysates by western blotting (Figure 2B). Except for *ACSL4*, all genes were upregulated at both the mRNA and protein levels in CDDP‐R tumors, suggesting an overall suppression of ferroptotic signaling in resistant tissues.

To further explore the link between PHB2 and ferroptosis, we conducted gene set enrichment analysis (GSEA) using the ferroptosis hallmark gene set on the TCGA stomach adenocarcinoma (STAD) cohort [32]. Tumors with high PHB2 expression exhibited significantly lower ferroptosis pathway activity, as measured by single‐sample GSEA (ssGSEA) scores (Figure 2C), indicating a negative association between PHB2 expression and ferroptosis signaling in human GC.

To functionally assess whether PHB2 regulates ferroptosis, we treated CDDP‐R AGS cells with a panel of cell death modulators: the ferroptosis inducer Erastin; ferroptosis inhibitors Ferrostatin‐1 and Liproxstatin‐1; and inhibitors targeting necroptosis (Necrostatin‐1), autophagy (3‐Methyladenine), and apoptosis (Z‐VAD‐FMK). Notably, only Ferrostatin‐1 and Liproxstatin‐1 significantly rescued cell death induced by PHB2 knockdown, whereas the other inhibitors had minimal or no effect (Figure 2D). These results strongly support a specific role for PHB2 in suppressing ferroptosis in cisplatin‐resistant GC cells.

Given that ferroptosis is driven by iron‐dependent lipid peroxidation and accumulation of oxidized phospholipids [33], we next assessed whether PHB2 regulates lipid peroxidation. PHB2 knockdown in CDDP‐R AGS cells significantly increased lipid peroxidation, as measured by Liperfluo, a fluorescent probe that detects lipid hydroperoxides (Figure 2E,F). This signal was further amplified upon Erastin treatment, consistent with enhanced ferroptotic activity (Figure 2F). Confocal microscopy using the ratiometric lipid peroxidation probe C11‐BODIPY revealed a marked increase in the oxidized form (C11^ox^, green) and a corresponding reduction in the non‐oxidized form (C11^Non‐ox^, red) following PHB2 silencing, which was further enhanced by Erastin co‐treatment (Figure 2G).

Lipid peroxidation proceeds in three phases‐initiation, propagation, and termination‐ultimately producing malondialdehyde (MDA), a stable end‐product and biomarker of lipid damage [34]. Consistent with this model, MDA levels were significantly elevated in PHB2‐depleted cells, particularly following Erastin exposure (Figure 2H). Furthermore, transmission electron microscopy (TEM) revealed classical ferroptotic ultrastructural changes in PHB2‐deficient cells, including reduced mitochondrial volume, cristae loss, and outer membrane rupture, features that were further exacerbated by Erastin (Figure 2I).

### 2.3 PHB2 Interacts With the AMP‐Binding Domain of ACSL3 to Suppress Ferroptosis

**FIGURE 3:**
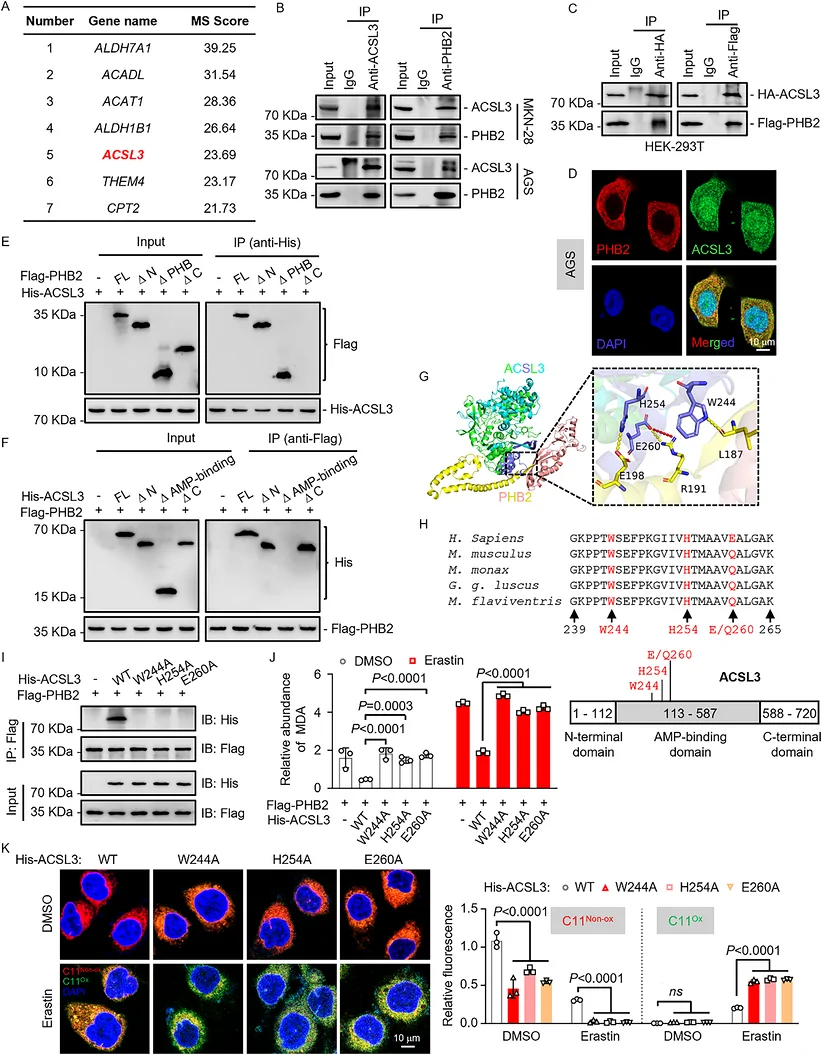
PHB2 suppresses ferroptosis by binding to the AMP‐binding domain of ACSL3. A) PHB2‐interacting enzymes identified by IP‐MS analysis, highlighting those involved in lipid metabolism and oxidative stress pathways in the table. B) Endogenous PHB2 and ACSL3 co‐immunoprecipitated in AGS and MKN‐28 cells. C) Co‐IP of exogenous Flag‐PHB2 and HA‐ACSL3 in HEK‐293T cells. D) Representative immunofluorescence images showing co‐localization of PHB2 and ACSL3 in AGS cells. Scale bar, 10 µm. E,F) Deletion‐mapping experiments revealed that the C‐terminal region of PHB2 is essential for binding to ACSL3 (E), while the AMP‐binding domain of ACSL3 is required for its interaction with PHB2 (F). G) Molecular docking of PHB2 into the structure of ACSL3, with both protein structures predicted using AlphaFold3. The overall complex is shown on the left, with the boxed region highlighting the predicted interaction interface between the two proteins. A close‐up view of key interacting residues is shown on the right, where PHB2 residues are colored in yellow and ACSL3 residues in purple/blue. Dashed lines represent predicted hydrogen bonds between the side chains of PHB2 and ACSL3. H) Conserved residues in ACSL3 (W244, H254, and E260) across different species are shown (Top). Schematic representation of ACSL3 domain architecture, highlighting the AMP‐binding domain (residues 113–587) and the positions of the predicted PHB2‐interacting residues (Bottom). I) Immunoblot (IB) analysis of anti‐Flag IPs and whole‐cell lysates (input) from HEK‐293T cells ectopically expressing Flag‐PHB2 and His‐tagged ACSL3 WT or indicated point mutants. W244, H254, and E260 are candidate PHB2‐interacting residues within the ACSL3 AMP‐binding domain. J,K) AGS cells overexpressing PHB2 along with either ACSL3 WT or point mutants were treated with DMSO (vehicle control) or Erastin. MDA levels (J) and the abundance of oxidized C11‐BODIPY (C11^ox^, green) (K) were subsequently measured. Data represent three independent experiments and are presented as mean ± SD. Quantitative data were normalized to the corresponding control (J,K). One‐way ANOVA test followed by Tukey's multiple comparisons (J,K).

Given PHB2's role in modulating lipid peroxidation during ferroptosis, we hypothesized that enzymes involved in lipid metabolism might interact with PHB2 to influence this process. To explore this possibility, we performed immunoprecipitation combined with mass spectrometry (IP‐MS) and identified several enzymes, including ALDH7A1, ACADL, ACAT1, ALDH1B1, ACSL3, THEM4, and CPT2, that participate in lipid metabolism and oxidative stress pathways (Figure 3A). Among these, ACSL3, a key enzyme in long‐chain fatty acyl‐CoA synthesis, emerged as a notable contributor to ferroptosis resistance in cancer cells [15]. Using both endogenous (Figure 3B) and exogenous (Figure 3C) co‐immunoprecipitation (Co‐IP) assays, we confirmed the interaction between PHB2 and ACSL3. Immunofluorescence staining revealed cytosolic co‐localization of PHB2 and ACSL3 in GC cells (Figure 3D), indicating spatial proximity conducive to protein‐protein interaction.

To define the molecular basis of the PHB2‐ACSL3 interaction, we generated truncated forms of both proteins and performed Co‐IP assays. These experiments demonstrated that the C‐terminal region of PHB2 is essential for binding to ACSL3 (Figure 3E), while the AMP‐binding domain of ACSL3 is necessary for its interaction with PHB2 (Figure 3F). Structural docking analysis further supported the PHB2‐binding capacity of ACSL3 (Figure 3G), revealing that several conserved ACSL3 residues (W244, H254, and E260) directly interact with PHB2 residues L187, E198, and R191, respectively (W244‐L187, H254‐E198, E260‐R191). Notably, all three ACSL3 residues are located within its AMP‐binding domain (Figure 3H), suggesting this region as a key interface for PHB2 engagement. Based on the docking model, we generated ACSL3 alanine substitution mutants (W244A, H254A, and E260A) to assess their impact on PHB2 binding. Mapping experiments showed that all three mutants exhibited reduced binding to PHB2 compared to the wild‐type (WT) ACSL3 (Figure 3I). Functionally, these mutations significantly increased MDA levels, especially in cells treated with Erastin (Figure 3J). Consistently, the abundance of oxidized C11 (C11^ox^, green) increased, while non‐oxidized C11 (C11^Non‐ox^, red) decreased, with Erastin treatment further amplifying these effects (Figure 3K). These site‐specific mutagenesis experiments highlight the functional importance of the PHB2‐ACSL3 interaction in regulating ferroptosis.

### 2.4 PHB2 Modulates ACSL3‐Dependent Phospholipid Remodeling to Control the MUFA‐To‐PUFA Ratio

**FIGURE 4:**
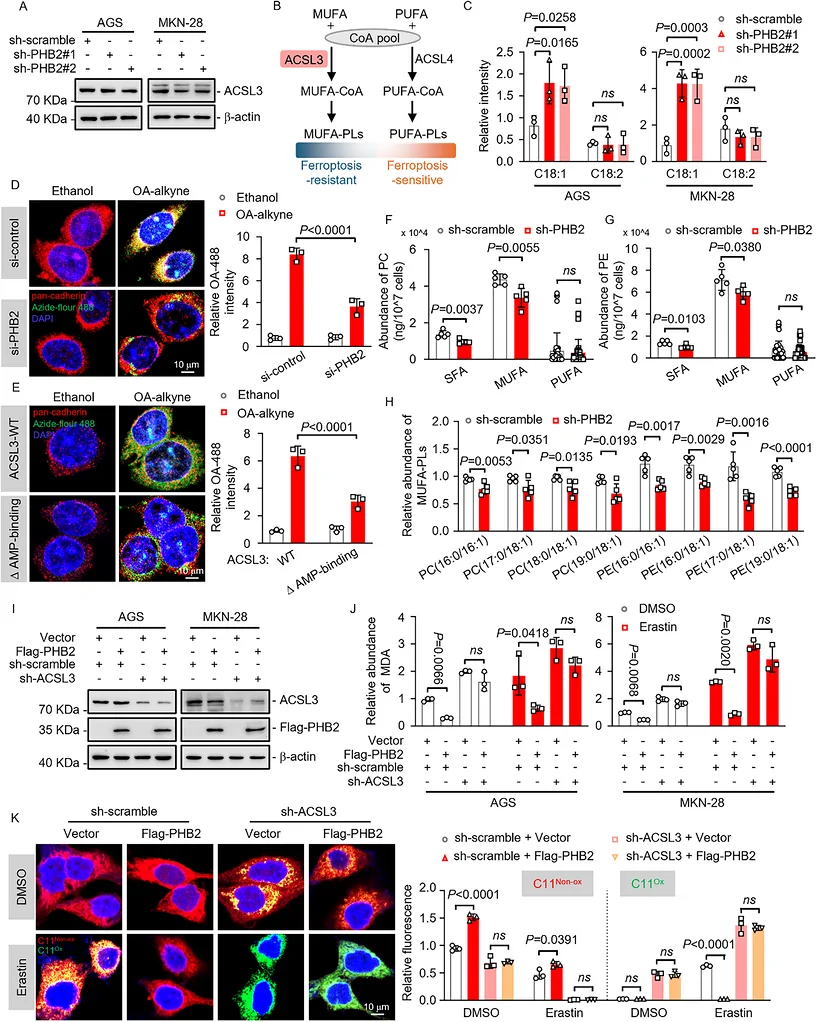
PHB2 regulates ACSL3 activities controlling the MUFA‐to‐PUFA ratio. A) Western blot analysis showing ACSL3 expression levels in AGS.sh‐PHB2 and MKN‐28.sh‐PHB2 cells. B) Schematic illustrating ACSL3‐mediated regulation of membrane phospholipid remodeling during ferroptosis. C) Quantification of cellular MUFA (OA; C18:1) and PUFA (LA; C18:2) levels by targeted lipidomics using LC‐MS/MS in AGS.sh‐PHB2 and MKN‐28.sh‐PHB2 cells (n = 3 independent samples). D) IF staining and quantification of membrane‐associated OA‐488 fluorescence intensity in AGS.si‐control and AGS.si‐PHB2 cells treated with either ethanol (vehicle control) or OA‐alkyne. Cells were treated with 20 µM OA‐alkyne for 10 hours, followed by a 10‐hour chase in regular medium. pan‐cadherin used as a plasma membrane marker. Scale bar, 10 µm. E) OA‐488 fluorescence intensity analysis revealed that the AMP‐binding domain of ACSL3, the key region for PHB2 binding, is required for ACSL3‐mediated incorporation of MUFA‐derived acyl chains into plasma membrane phospholipids. Scale bar, 10 µm. F,G) Untargeted lipidomics analysis of SFAs, MUFAs, and PUFAs levels in PC (F) and PE (G) in AGS cells treated with sh‐PHB2. PC, phosphatidylcholine; PE, phosphatidylethanolamine. H) Alterations in the levels of specific PL species in AGS cells upon PHB2 knockdown. I) Western blot analysis assessed the efficiency of PHB2 overexpression and ACSL3 knockdown in AGS and MKN‐28 cells. J,K) The subsequent MDA assay (J) and BODIPY 581/591 C11 staining (K) revealed that PHB2 overexpression reduced lipid peroxidation levels, which were rescued by ACSL3 silencing in both AGS and MKN‐28 cells, regardless of DMSO (vehicle control) or Erastin treatment. Data represent three independent experiments and are presented as mean ± SD. Quantitative data were normalized to the corresponding control (C–E,H,J,K). Two‐tailed Student's *t*‐test (F‐H). One‐way ANOVA followed by Tukey's multiple comparison (C–E,J,K).

To explore how the PHB2‐ACSL3 interaction contributes to ferroptosis resistance in GC, we first examined ACSL3 expression following PHB2 knockdown. PHB2 knockdown did not alter ACSL3 protein and mRNA levels (Figure 4A; Figure S3A), suggesting that PHB2 may regulate ACSL3 function rather than its expression. Given that ACSL3 catalyzes the activation of monounsaturated fatty acids (MUFAs) into MUFA‐CoAs, which serve as substrates for phospholipid remodeling [15], we next investigated whether PHB2 affects ACSL3‐dependent MUFA utilization (Figure 4B). We hypothesized that PHB2‐mediated activation of ACSL3 promotes MUFA‐CoA formation and facilitates the incorporation of MUFAs into membrane phospholipids, thereby modulating acyl‐CoA availability and reducing membrane susceptibility to lipid peroxidation.

To assess ACSL3 activity, we measured the levels of MUFA (oleic acid [OA], C18:1) and PUFA (linoleic acid, C18:2) in AGS.sh‐PHB2 and MKN‐28.sh‐PHB2 cells. Notably, PHB2 knockdown significantly increased MUFA levels without affecting PUFA abundance, indicating that PHB2 enhances ACSL3 activity (Figure 4C). To trace MUFA utilization, we pulse‐labeled cells with OA‐alkyne and performed Click Chemistry with azide‐Fluor 488 to visualize MUFA‐derived acyl chain incorporation into membrane phospholipids. OA‐alkyne incorporation was detected through copper(I)‐catalyzed alkyne‐azide cycloaddition with azide‐fluor 488, producing the fluorescent OA‐488 conjugate [16]. PHB2 knockdown markedly reduced membrane‐associated OA‐488 fluorescence (yellow signal), indicating impaired MUFA‐PL synthesis, likely due to diminished ACSL3 activity (Figure 4D). Moreover, disruption of the ACSL3 AMP‐binding domain, the key PHB2‐interacting region, abolished MUFA‐derived acyl chain incorporation into membrane phospholipids, underscoring its essential role in ACSL3 enzymatic activity (Figure 4E).

To further assess whether PHB2 depletion affects CoA‐dependent fatty acid activation, we performed targeted metabolomics to profile total acyl‐CoA species in control and PHB2‐knockdown cells. PHB2 knockdown broadly remodeled the acyl‐CoA landscape, with reduced levels of several MUFA‐CoA species, including 14:1‐CoA, 15:1‐CoA, 16:1‐CoA, 18:1‐CoA, and 20:1‐CoA (Figure S3B). These results indicate that PHB2 depletion perturbs fatty acyl‐CoA homeostasis and CoA‐dependent fatty acid activation, consistent with altered fatty acyl‐CoA availability. We then examined phospholipid composition in membrane fractions of parental and PHB2‐knockdown GC cells using untargeted lipidomics. A total of 43 lipid species spanning five major PL classes (PC, PE, PG, PI, and PS) were identified. Compared with controls, PHB2 knockdown reduced overall lipid content (Figure S3C) and decreased most lipid species to varying degrees, although some changes were not statistically significant (Figure S3D). In mammalian cells, saturated fatty acids (SFAs) are predominantly located at the sn‐1 position of PLs, while unsaturated fatty acids occupy the sn‐2 position [35]. We therefore focused on analyzing unsaturated fatty acids at the sn‐2 position. In AGS.sh‐PHB2 cells, PC and PE species containing SFAs and MUFAs were significantly reduced compared with sh‐scramble controls, while PC and PE species containing PUFAs remained unchanged (Figure 4F,G). This strong association between MUFA‐containing PC/PE and PHB2 expression prompted further analysis of long‐chain MUFA‐containing PLs (LC‐MUFAs). Several representative LC‐MUFA‐containing PLs, including PC (16:0/16:1), PE (16:0/16:1), PE (17:0/18:1), and PE (19:0/18:1), were significantly decreased in the PHB2 knockdown cells (Figure 4H), while LC‐PUFA‐containing PLs were unaffected (Figure S3E).

These findings indicate that PHB2 contributes to membrane phospholipid remodeling by enhancing ACSL3 activity. To further confirm this mechanism, we overexpressed PHB2 in AGS.sh‐ACSL3 and MKN‐28.sh‐ACSL3 sublines (Figure 4I). Lipid peroxidation assays showed that PHB2 overexpression significantly reduced MDA levels and oxidized C11 (C11^ox^, green), particularly under Erastin treatment, and that these effects were abolished by ACSL3 knockdown (Figure 4J,K). Collectively, these results suggest that PHB2 regulates the MUFA‐to‐PUFA ratio by enhancing ACSL3 activity, thereby reshaping membrane phospholipid composition and reducing susceptibility to lipid peroxidation.

### 2.5 Mavorixafor Disrupts the PHB2‐ACSL3 Interaction to Overcome Ferroptosis Resistance

**FIGURE 5:**
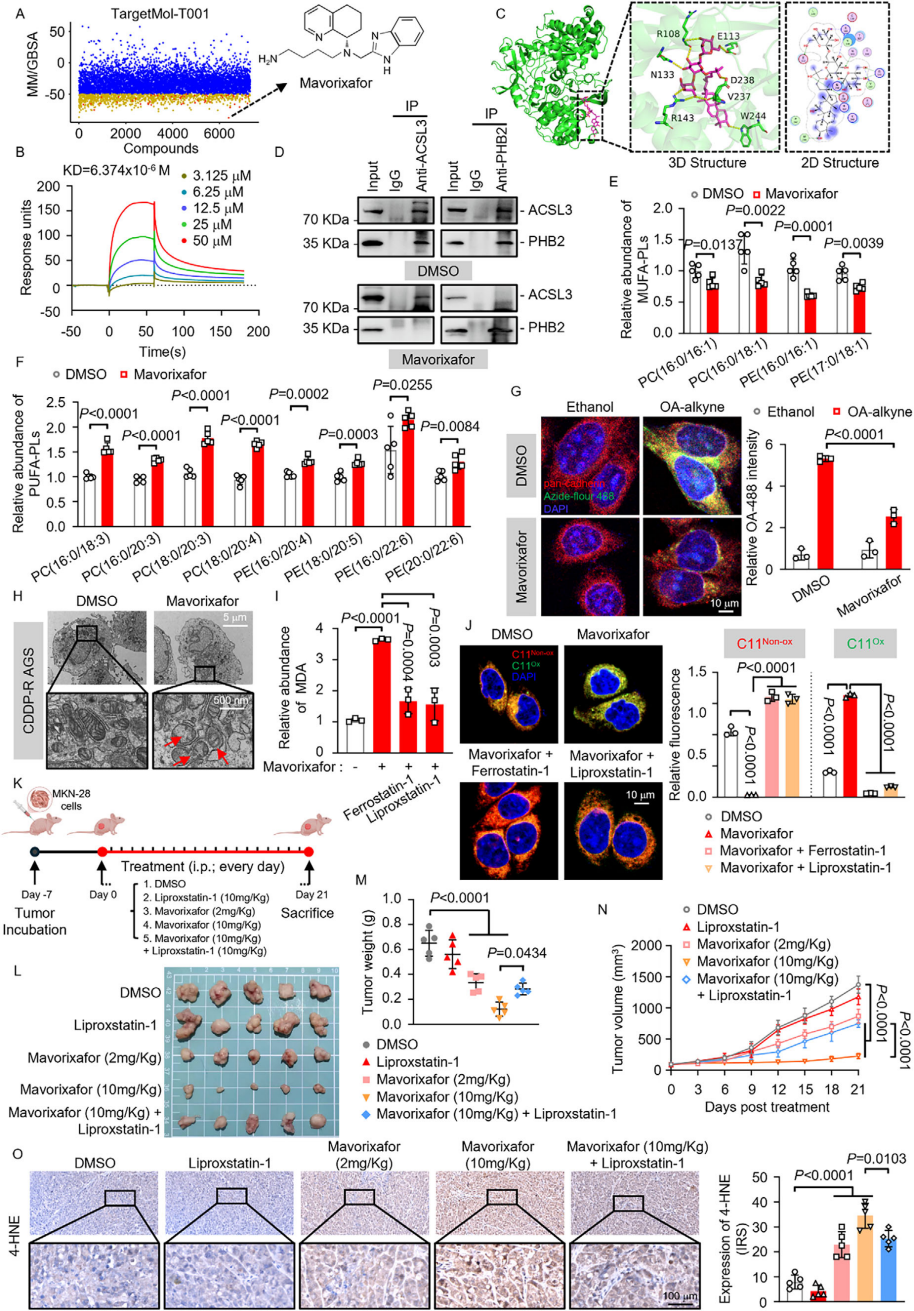
Mavorixafor targets the PHB2‐ACSL3 interaction to regulate ACSL3‐dependent phospholipid remodeling and overcome ferroptosis resistance in GC. A) Virtual screening of the TargetMol‐T001 library, ranked by MMGBSA binding free energy, identified Mavorixafor as a potential inhibitor of the PHB2‐ACSL3 interaction. The chemical structure of Mavorixafor is shown on the right. B) Surface plasmon resonance (SPR) analysis of the binding affinity between immobilized recombinant human ACSL3 and serially diluted Mavorixafor (KD = 6.374×10^−6^ M). C) Binding conformation of compound Mavorixafor with ACSL3. Overall view (left), local 3D view (middle), and local 2D view (right) of the Mavorixafor‐ACSL3 complex. Carbon atoms of Mavorixafor are shown in magenta, oxygen atoms in red, and nitrogen atoms in blue. ACSL3 is represented as a green cartoon structure. Yellow dashed lines indicate hydrogen bonds, while blue and green arrows represent hydrogen bond interactions in the 2D view. D) Co‐IP assays demonstrated that Mavorixafor disrupts the interaction between PHB2 and ACSL3 in AGS cells. E,F) Untargeted lipidomics analysis revealed that Mavorixafor treatment significantly reduced the abundance of MUFA‐PLs (E), while increasing the levels of PUFA‐PLs (F) in AGS cells. G) OA‐488 fluorescence analysis revealed that Mavorixafor treatment impaired the incorporation of MUFA‐derived acyl chains into plasma membrane phospholipids. Scale bar, 10 µm. H) TEM images showing mitochondrial morphology and cristae structure in CDDP‐R AGS cells treated with DMSO or Mavorixafor. Scale bar, 5 µm (top) and 500 nm (bottom). I,J) MDA assay (I) and BODIPY 581/591 C11 staining (J) demonstrated that Mavorixafor treatment elevated lipid peroxidation levels in AGS cells, which were reversed by the ferroptosis inhibitors Ferrostatin‐1 and Liproxstatin‐1. K) Schematic diagram of treatment groups in the MKN‐28 xenograft tumor model (n = 5 per group). L–N) Representative images (L), tumor weight (M), and growth curves (N) of MKN‐28 xenografts treated with DMSO, Liproxstatin‐1, Mavorixafor (2 mg/kg or 10 mg/kg), or a combination of Mavorixafor and Liproxstatin‐1 in BALB/c nude mice. Scale bar, 1 cm. O) IHC staining and quantification of 4‐HNE in MKN‐28 xenograft tumors with the indicated treatments. Representative IHC images are shown. Scale bar, 100 µm. IRS: Immunoreactive score. Data represent three independent experiments and are presented as mean ± SD. Quantitative data were normalized to the corresponding control (E–G,I,J). Two‐tailed Student's *t*‐test (E,F). One‐way (G,I,J,M,O) or two‐way ANOVA followed by Tukey's multiple comparison (N).

Given the critical role of the PHB2‐ACSL3 interaction in ferroptosis resistance, we conducted molecular docking analysis to identify potential drugs targeting this interaction. A total of 7,312 compounds from the TargetMol‐T001 library were screened to select the most promising candidates (Figure 5A; Figure S4A). The ACSL3 binding pocket, highlighted in the black box, was selected for virtual screening based on its interaction with PHB2 (Figure S4B–D). To refine the selection, we performed molecular docking using the Glide SP (Standard Precision) approach, followed by MMGBSA (Molecular Mechanics Generalized Born Surface Area) analysis on the top 10% of conformations (Figure S4E–G). The screened compounds were further evaluated through protein‐ligand interaction fingerprint (PLIF) analysis (Figure S4H) and structural diversity analysis (Figure S4I,J). Subsequently, the top 50 compounds were selected based on binding free energy and subjected to single‐concentration (50 µM) surface plasmon resonance (SPR) screening (Table S2). We excluded peptides due to their poor stability, permeability, and bioavailability [36] and evaluated the top three candidates (Mavorixafor, Isoliensinine, and GMB‐475) using five drug concentration gradients to determine their binding affinity (K_D) with ACSL3 (Figure 5B; Figure S4K,L). Mavorixafor emerged as a potent ACSL3 inhibitor that effectively blocked its interaction with PHB2 (Figure 5A). Binding mode analysis identified key ACSL3 residues involved in this interaction, all of which are located within the AMP‐binding domain (Figure 5C). Finally, endogenous Co‐IP confirmed that Mavorixafor disrupted the PHB2‐ACSL3 complex, supporting its potential role in overcoming ferroptosis resistance (Figure 5D). Importantly, Mavorixafor did not alter ACSL3 protein levels in control or PHB2‐knockdown AGS and MKN‐28 cells, indicating that its effect is not expression‐dependent (Figure S4M,N). Moreover, ACSL3 depletion and PHB2 knockdown both attenuated Mavorixafor‐mediated growth inhibition, supporting that Mavorixafor acts mainly through the PHB2–ACSL3 interaction rather than through PHB2 or ACSL3 alone (Figure S4O,P).

We then examined whether Mavorixafor regulates membrane phospholipid remodeling during ferroptosis. Untargeted lipidomics analysis revealed that Mavorixafor treatment in AGS cells significantly reduced LC‐MUFA‐containing PLs, such as PC (16:0/16:1), PC (16:0/18:1), PE (16:0/16:1), and PE (17:0/18:1) (Figure 5E), while increasing LC‐PUFA–containing PLs, including PC (16:0/18:3), PC (18:0/20:3), PE (16:0/20:4), and PE (16:0/22:6) (Figure 5F), suggesting a shift in membrane phospholipid composition potentially mediated by ACSL3 activity. Consistently, OA‐alkyne incorporation analysis demonstrated a significant reduction in OA‐488 fluorescence intensity following Mavorixafor treatment (Figure 5G). Furthermore, TEM analysis revealed Mavorixafor‐induced alterations in mitochondrial morphology in CDDP‐R AGS cells (Figure 5H).

Building on Mavorixafor's impact on membrane phospholipid remodeling, we treated AGS cells with ferroptosis inhibitors (Ferrostatin‐1 and Liproxstatin‐1) and found that they significantly rescued Mavorixafor‐induced membrane lipid peroxidation, as evidenced by reduced MDA levels (Figure 5I) and decreased oxidized C11 (C11^ox^, green) abundance (Figure 5J). These findings suggest that Mavorixafor may promote membrane lipid peroxidation through ACSL3‐dependent phospholipid remodeling.

To assess the effects of Mavorixafor on malignant behaviors in GC in vivo, BALB/c nude mice bearing MKN‐28 xenograft tumors were treated with DMSO, Liproxstatin‐1, Mavorixafor (2 mg/kg or 10 mg/kg), or a combination of Mavorixafor and Liproxstatin‐1 (Figure 5K). Liproxstatin‐1 alone had no significant effect on tumor growth, while Mavorixafor inhibited tumor growth in a dose‐dependent manner. Notably, Liproxstatin‐1 reversed the tumor‐suppressive effect of Mavorixafor in GC xenografts (Figure 5L–N). IHC analysis revealed that Liproxstatin‐1 treatment attenuated Mavorixafor‐induced accumulation of 4‐HNE–stained lipid peroxidation products in GC xenograft tumors (Figure 5O). Our findings demonstrate that Mavorixafor targets the PHB2‐ACSL3 interaction to overcome ferroptosis resistance by regulating ACSL3‐dependent phospholipid remodeling in GC.

### 2.6 Mavorixafor Increases Drug Sensitivity in CDXs and PDOs in a Ferroptosis‐Dependent Manner

**FIGURE 6:**
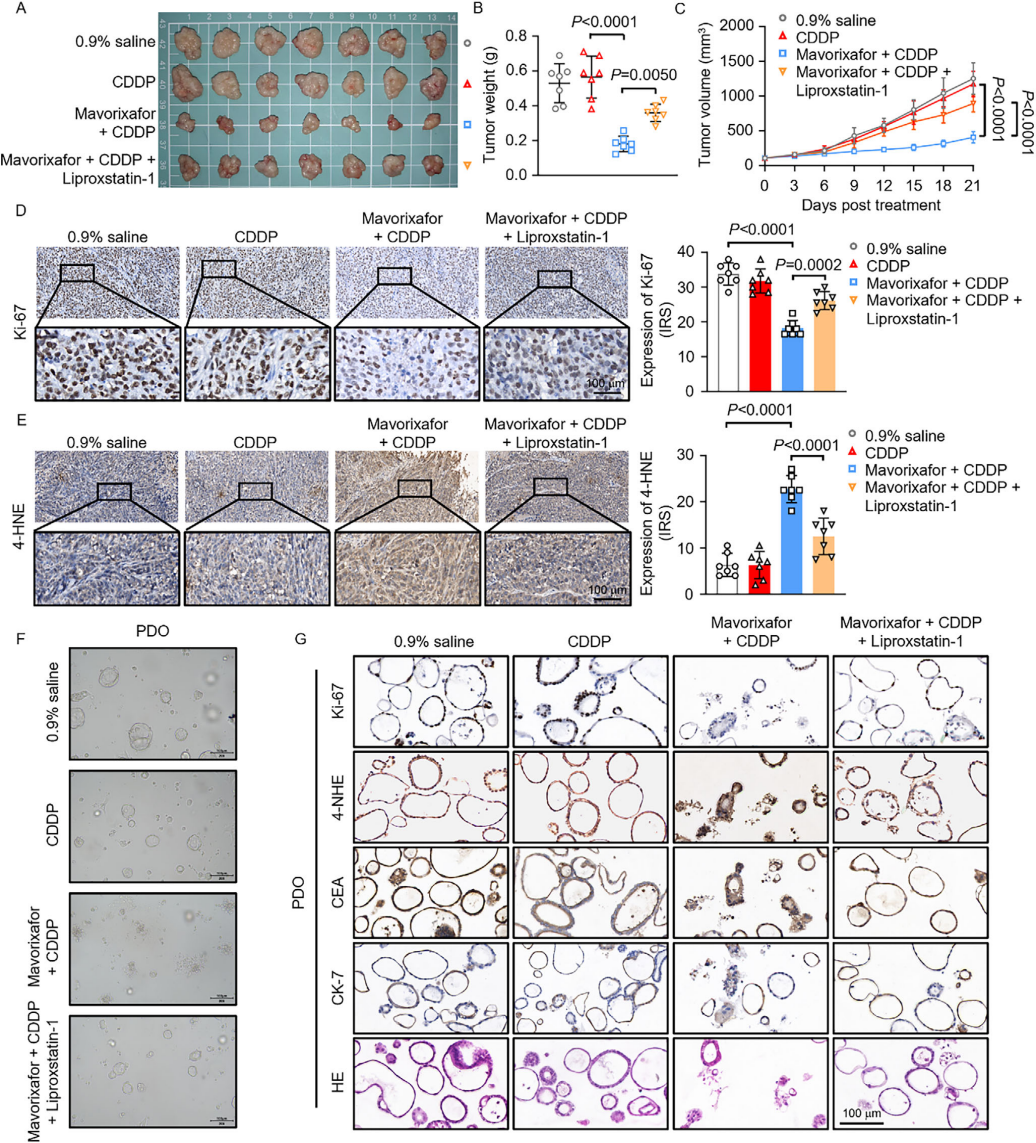
Mavorixafor sensitizes drug‐resistant GC via ferroptosis in CDX and PDO models. A–C) Representative image (A), tumor weight (B), and growth curves (C) of CDDP‐R AGS xenografts treated with 0.9% saline (control), CDDP, Mavorixafor combined with CDDP, and Mavorixafor combined with CDDP and Liproxstatin‐1 in BALB/c nude mice. Scale bar, 1 cm. D, E) IHC staining and quantification of Ki‐67 (D) and 4‐HNE (E) in CDDP‐R AGS xenograft tumors with the indicated treatments. Representative IHC images are shown. Scale bar, 100 µm. IRS: Immunoreactive score. F) Representative bright‐field images of CDDP‐R GC PDOs treated with 0.9% saline (control), CDDP, Mavorixafor combined with CDDP, and Mavorixafor combined with CDDP and Liproxstatin‐1. G) Representative IHC staining of Ki‐67, 4‐HNE, CEA, and CK‐7, as well as HE staining, in CDDP‐R GC PDOs under the indicated treatments. Scale bar, 100 µm. Data represent three independent experiments and are presented as mean ± SD. One‐way (B,D,E) or two‐way ANOVA followed by Tukey's multiple comparison (C).

To validate the therapeutic potential of Mavorixafor in suppressing tumor growth in vivo, we established two mouse models: an orthotopic cell line‐derived xenograft (CDX) model and an orthotopic patient‐derived organoid (PDO) model. In the CDDP‐R AGS xenograft model, we evaluated the effects of different treatments by dividing mice into four groups: 0.9% saline (control), CDDP, Mavorixafor combined with CDDP, and Mavorixafor combined with CDDP and Liproxstatin‐1 (Figure S5A). As expected, CDDP alone had minimal impact on tumor growth. However, Mavorixafor significantly enhanced tumor sensitivity to CDDP, leading to greater tumor suppression. Notably, this effect was reversed when Liproxstatin‐1 was added, indicating that Mavorixafor's ability to enhance CDDP sensitivity is ferroptosis‐dependent (Figure 6A–C). In addition, IHC analysis showed that Liproxstatin‐1 treatment mitigated the effects of Mavorixafor on CDDP sensitivity, as indicated by the partial restoration of Ki67‐positive proliferating cells (Figure 6D) and a reduction in 4‐HNE‐stained lipid peroxidation products (Figure 6E). These findings further reinforce that Mavorixafor enhances CDDP sensitivity through a ferroptosis‐dependent mechanism. To assess potential systemic toxicity, we measured serum liver and kidney function markers at the experimental endpoint. Serum alanine aminotransferase (ALT), aspartate aminotransferase (AST), blood urea nitrogen (BUN), and creatinine (Cr) levels showed only minor intergroup variations and remained within the expected physiological range, indicating that the combination treatment did not cause obvious hepatic or renal toxicity in mice (Figure S5B–E).

To further validate these findings in a clinically relevant setting, we applied the same treatment regimens in a CDDP‐R GC PDO model. Consistent with the xenograft results, Mavorixafor markedly enhanced tumor sensitivity to CDDP, leading to a substantial reduction in tumor burden. This effect, however, was abrogated by Liproxstatin‐1, which restored tumor growth (Figure 6F). IHC analysis further confirmed that Liproxstatin‐1 mitigated Mavorixafor‐induced ferroptosis, as reflected by increased Ki67‐positive cells (a marker of cell proliferation) and diminished 4‐HNE accumulation (a marker of lipid peroxidation and ferroptosis) (Figure 6G). In addition, CEA and CK‐7 staining verified the gastric epithelial origin of the PDOs, supporting the clinical relevance of this model (Figure 6G). These findings reinforce the ferroptosis‐dependent mechanism by which Mavorixafor enhances CDDP sensitivity, highlighting its potential as a therapeutic strategy for drug‐resistant GC.

## 3 Discussion

Therapeutic resistance remains a major challenge in GC, and ferroptosis evasion has emerged as a critical yet incompletely understood driver of treatment failure [6, 7, 8, 37]. In this study, we uncover a previously unrecognized lipid metabolic mechanism underlying ferroptosis resistance in GC. We demonstrate that PHB2 directly binds to the AMP‐binding domain of ACSL3 and enhances its MUFA‐CoA synthetase activity. This increases MUFA incorporation into phospholipids and skews the MUFA‐to‐PUFA ratio toward a ferroptosis‐resistant membrane state. This phospholipid remodeling enables cancer cells to evade lipid peroxidation and ferroptotic death under chemotherapeutic stress. Disrupting the PHB2‐ACSL3 interaction with Mavorixafor [26], a clinically available CXCR4 antagonist with no prior link to ferroptosis, selectively reduces MUFA‐enriched phospholipids and restores PUFA content. This re‐sensitizes platinum‐resistant tumors to cisplatin in both CDX and PDO models.

Most ferroptosis studies to date have focused on antioxidant defense systems such as GPX4, FSP1, and SLC7A11, or on iron metabolism [30, 38, 39, 40, 41]. In contrast, our work highlights a distinct regulatory axis in which protein‐protein interaction modulates lipid metabolic enzyme activity to remodel membrane composition. While ACSL3 has been implicated in MUFA‐dependent ferroptosis protection [15], the mechanisms governing its enzymatic specificity have remained unclear. Our data provide mechanistic evidence that PHB2, traditionally recognized for roles in mitochondrial homeostasis and mitophagy [18, 19], can act in the cytosol to allosterically enhance ACSL3 activity, revealing a moonlighting function for PHB2 in ferroptosis regulation. This mechanism‐centered view adds a new dimension to the lipid metabolism‐ferroptosis interface.

We further identify Mavorixafor as a first‐in‐class inhibitor of the PHB2‐ACSL3 complex. Although Mavorixafor is clinically established as a CXCR4 antagonist, the mechanistic evidence obtained in this study supports the PHB2–ACSL3 complex as a major functional target within the concentration range examined. Mavorixafor directly bound ACSL3, disrupted its interaction with PHB2, and its growth‐inhibitory effects were attenuated by depletion of either PHB2 or ACSL3. These observations suggest that the ferroptosis‐sensitizing activity identified here is mediated predominantly through PHB2–ACSL3‐dependent lipid remodeling. Unlike conventional ferroptosis inducers that broadly inhibit GPX4 or perturb iron homeostasis [29, 42], Mavorixafor selectively targets the enzymatic regulation of lipid remodeling, potentially reducing systemic toxicity. Its favorable pharmacokinetic and safety profile, already established in clinical use, positions it as a rapidly translatable candidate for overcoming chemoresistance [26, 43]. The efficacy of Mavorixafor was robustly validated in orthotopic CDDP‐resistant GC CDX and PDO models, with ferroptosis dependency confirmed by rescue with Liproxstatin‐1.

Our results also reveal an intriguing divergence between genetic PHB2 knockdown and pharmacologic disruption with Mavorixafor. While both reduce MUFA abundance, PHB2 depletion broadly impairs fatty acid processing and mitochondrial lipid metabolism, whereas Mavorixafor selectively restores PUFA‐containing phospholipids without overt mitochondrial damage. This suggests that targeted protein–protein interaction disruption may achieve ferroptosis sensitization with greater metabolic precision. By sparing the broader metabolic functions of PHB2 while disrupting its interaction with ACSL3, this strategy may reduce off‐target metabolic perturbations and improve tolerability compared with global PHB2 inhibition or depletion. Given that lipid remodeling contributes to therapy resistance in multiple malignancies [44], including prostate and breast cancers, this concept warrants exploration beyond GC.

Several important questions remain. Although ACSL3‐driven MUFA activation contributes to CoA‐dependent lipid remodeling, steady‐state acyl‐CoA levels are shaped by fatty acid activation, downstream utilization, and compensatory remodeling. Thus, PHB2 loss may reduce MUFA‐CoA formation and broadly rewire fatty acyl‐CoA availability rather than produce a simple reciprocal shift between MUFA‐CoA and PUFA‐CoA species, thereby contributing to a state of disordered acyl‐CoA homeostasis. Because extracellular CoA is poorly membrane‐permeable, future studies using more tractable approaches to modulate intracellular CoA availability may further clarify the contribution of CoA‐pool dynamics to PHB2–ACSL3‐mediated lipid remodeling. The universality of the PHB2‐ACSL3 axis across cancer types, the structural basis for PHB2‐mediated ACSL3 activation, and the potential immunomodulatory consequences of ferroptosis induction in an intact tumor microenvironment merit further investigation. Additionally, time‐resolved lipidomics in in vivo models could clarify the causal sequence linking MUFA‐to‐PUFA ratio shifts to ferroptotic commitment. Structure‐activity studies of Mavorixafor analogs may enable optimization for oncology use.

Collectively, our findings establish PHB2‐dependent activation of ACSL3 as a critical ferroptosis resistance mechanism in GC and provide preclinical evidence supporting Mavorixafor as a repurposable agent to restore ferroptotic vulnerability in chemoresistant tumors. Targeting lipid remodeling through inhibition of protein‐protein interactions offers a conceptually distinct and potentially safer route for precision ferroptosis‐based cancer therapy. By revealing a druggable metabolic vulnerability, our work paves the way for ferroptosis restoration to become a clinically actionable strategy against therapy‐refractory cancers.

## 4 Materials and Methods

### 4.1 Ethics Statement 

All experiments were conducted in compliance with institutional and national ethical standards. Animal studies and the use of human tissue samples were approved by the Institutional Animal Care and Ethics Committee of Anhui Medical University (Approval No. 81250111 and LLSC20250591). Written informed consent was obtained from all human participants before sample collection, and no financial compensation was provided. Specimens were processed and analyzed in accredited research laboratories.

### 4.2 Cell Culture and Human Tissue

The Human GC cell lines AGS and MKN‐28, as well as the HEK293T human embryonic kidney cell line, were obtained from the Institute of Biochemistry and Cell Biology, Shanghai Institutes for Biological Sciences, Chinese Academy of Sciences. A CDDP‐R AGS cells (Cat# KGG3506‐1) were purchased from Jiangsu KeyGEN BioTECH Corp., Ltd. AGS and its drug‐resistant counterpart were cultured in F‐12 HAM'S medium (HyClone, USA), while MKN‐28 cells were maintained in RPMI‐1640 (HyClone, USA). All media were supplemented with 10% fetal bovine serum (Gibco, USA) and 1% penicillin‐streptomycin. All cells were incubated at 37°C in a humidified atmosphere containing 5% CO_2_. Cell lines were routinely tested for Mycoplasma contamination every three months and authenticated by short tandem repeat (STR) profiling. GC tissue samples were collected from CDDP‐S (n = 10) and CDDP‐R (n = 24) patients at the Department of Gastrointestinal Surgery, The Second Affiliated Hospital of Anhui Medical University (Hefei, China).

### 4.3 Cell Transfection and Infection

Full‐length and truncated forms of human PHB2 and ACSL3, as well as ACSL3 point mutants (W244A, H254A, and E260A), were cloned into the p3xFLAG‐Myc‐CMV‐24 and pcDNA3.1(+)‐myc‐His expression vectors. Short hairpin RNAs (shRNAs) targeting PHB2 and ACSL3 were inserted into the GV248 lentiviral vector (GeneChem), and lentivirus processing and infection were performed according to the manufacturer's instructions. Transfection of small interfering RNAs (siRNAs) or plasmids was carried out using Lipofectamine 3000 (Thermo Fisher Scientific), following the manufacturer's protocol. A complete list of siRNAs and shRNAs is provided in Table S1.

### 4.4 Cell Viability Assay

For the MTT assay, 4 × 10^^3^ cells were seeded in each well of a 96‐well plate with 200 µl of culture medium and incubated for 96 hours. Subsequently, 20 µl of MTT solution (5 mg/ml) (BioFroxx) was added, and the cells were incubated for an additional 4 hours. Absorbance was measured at 490 nm using a microplate reader (USCN KIT IN).

For the colony formation assay, 2 × 10^^3^ cells were seeded in a 6‐well plate and cultured with media changes every 3 days. After 2 weeks, cells were fixed with methanol, stained with 0.1% crystal violet (Beyotime) for 30 minutes, and colonies were counted using ImageJ.

### 4.5 Immunofluorescence (IF) Staining

Briefly, cells were fixed with methanol at 4°C for 30 minutes, then incubated overnight at 4°C with anti‐PHB2 mouse antibodies (1:50, Proteintech) and anti‐ACSL3 rabbit antibodies (1:50, Proteintech). After blocking with goat serum at room temperature (RT) for 30 minutes, the cells were incubated for 2 hours with Alexa Fluor 488 anti‐rabbit IgG (1:200, Proteintech) and Alexa Fluor 594 anti‐mouse IgG secondary antibodies (1:200, Proteintech). Nuclei were stained with 4′,6‐diamidino‐2‐phenylindole (DAPI) (Beyotime), and images were captured using a confocal microscope (Carl Zeiss, LSM880).

### 4.6 Immunoprecipitation (IP) and Immunoblot Analysis

IP was conducted following established protocols [45]. Briefly, cell lysates were incubated with the specified antibody and protein A/G agarose beads (Thermo Fisher Scientific) at 4°C to facilitate complex formation. The bound proteins were then eluted by heating in SDS buffer, separated by SDS‐PAGE, and analyzed by immunoblotting as previously described.

### 4.7 Transmission Electron Microscopy (TEM)

TEM was performed at the Sci‐go Instrument Testing Platform. Samples were fixed in 2.5% glutaraldehyde (Sigma) in 0.1 M cacodylate buffer (Sigma) (pH 7.3), rinsed with the same buffer, and dehydrated through a graded ethanol (Sigma) series (30%, 50%, 70%, 80%, 90%, 95%). Specimens were then infiltrated and embedded in LX‐112 resin, polymerized at 70°C overnight. Ultrathin sections (70–90 nm) were prepared using an Ultracut microtome (LEICA UC7), stained with lead citrate and 50% ethanol‐saturated uranyl acetate for 5–10 minutes each, and examined on a HT7700 TEM (Hitachi) at 80 kV. Digital images were acquired using the microscope's integrated high‐resolution CCD camera.

### 4.8 Lipid Peroxidation Malondialdehyde (MDA) Assay

Cells were pre‐treated and incubated with the indicated compounds for defined time periods. Following treatment, cells were harvested by trypsinization, washed with PBS, and lysed in immunoprecipitation (IP) buffer for 30 minutes on ice. Lysates were then centrifuged at 3000 × g for 10 minutes at 4 °C, and the supernatants were collected for analysis. Preparation of the TBA storage solution and MDA working solution was performed according to the manufacturer's instructions (Beyotime). MDA levels were determined by measuring absorbance at 532 nm using a microplate reader. MDA content (nmol/mg protein) was calculated using the formula: MDA (nmol/mg) = (OD × V_total) / (ε × d × C_pr × V_sample) × 10^^9^, where: *V_total* is the total reaction volume, *ε* is the molar extinction coefficient of MDA (155 × 10^^3^ L/mol/cm), *d* is the path length of the 96‐well plate, *C_pr* is the protein concentration of the sample, and *V_sample* is the volume of the sample used. All MDA values were normalized to total protein content to ensure consistency across samples.

### 4.9 Lipid Peroxidation BODIPY 581/591 C11 Assay

Pre‐treated 2 × 10^^4^ cells were seeded into each well of a 24‐well plate containing a sterile coverslip and incubated overnight. The following day, cells were washed with PBS and incubated with 5 µM BODIPY 581/591 C11 (Thermo Fisher Scientific) in serum‐free culture medium for 1.5 hours at 37°C. After incubation, cells were fixed with methanal for 30 minutes at 4°C and subsequently stained with DAPI. Coverslips were then mounted on glass slides, and samples were imaged using a confocal laser scanning microscope. Image analysis and fluorescence quantification were performed using ImageJ software.

### 4.10 Lipid Peroxide (Liperfluo) Detection Assay

4 × 10^^5^ cells were seeded into each well of a 6‐well plate with 2 mL of culture medium and incubated overnight. The supernatant was then removed, and the cells were washed once with serum‐free culture medium. A 2 µmol/L Liperfluo working solution was prepared according to the manufacturer's instructions (DOJinDO). 1 mL of the Liperfluo working solution was added to each well, and the cells were incubated at 37°C for 1.5 hours. The final concentration of Liperfluo was optimized based on experimental conditions. After incubation, the supernatant was removed, and the cells were washed twice with serum‐free culture medium. The cells were then analyzed using flow cytometry (BD Biosciences).

### 4.11 Alkyne Labelling, Imaging and Quantification

Pre‐treated 2 × 10^^4^ cells were seeded into each well of a 24‐well plate containing a coverslip and incubated overnight. The following day, cells were washed once with PBS and treated for 10 hours with or without 20 µM OA‐alkyne (Cayman Chemical) in culture medium. After treatment, cells were washed three times with PBS and fixed with methanol for 30 minutes at 4°C. Subsequently, a click reaction solution containing 0.1 mm Azide‐Fluor 488 (Sigma), 1 mM copper sulfate (AbMole BioScience), and 1 mM Tris(2‐carboxyethyl) phosphine hydrochloride (Sigma) in PBS was added to the cells and incubated for 1 hour at RT in a dark, humid chamber. After the reaction, cells were blocked with PBS‐BT (1× PBS, 3% BSA, 0.1% Triton X‐100, and 0.02% NaN_3_) for 1 hour at RT, followed by incubation with primary antibody for 4 hours at 4°C. The cells were then washed with PBS‐BT, incubated with secondary antibody for 2 hours at 4°C, and counterstained with DAPI. Finally, samples were mounted and imaged using a confocal laser scanning microscope. Image quantification of the OA‐alkyne experiments was performed using ImageJ software.

### 4.12 Free Fatty Acid Quantification

The concentrations of C18:1 and C18:2 were quantified in three independent samples of AGS.sh‐PHB2 and MKN‐28.sh‐PHB2 using LC‐MS/MS. 3 × 10^^5^ cells were seeded into 10 cm dishes and incubated at 37°C for 24 hours. Cells were then washed with PBS and incubated in F12 medium containing 100 µM Oleic acid‐^13^C_18_ (Sigma) and 100 µM ^2^H‐Linoleic acid (MedChemExpress) for 5 days. After incubation, 1 × 10^^7^ cells were collected by centrifugation at 3500 rpm, and the supernatant was removed. The cell pellet was resuspended in 400 µL of Mixture A (acetonitrile:methanol:water, 2:2:1), then centrifuged at 1000 × g for 5 minutes. The lower organic phase was transferred to a new 1.5 mL EP tube. The remaining upper phase was mixed with 50 µL formic acid (Sigma) and 1 mL Mixture A, vortexed for 1 minute, and centrifuged at 8000 rpm at 4°C for 10 minutes. The lower organic phase was collected, combined with the first extract, and dried under nitrogen for 2 hours. The residue was re‐dissolved in 100 µL of Mixture B (methanol:acetonitrile, 2:1) for Liquid Chromatography with tandem mass spectrometry (LC‐MS/MS) analysis in MRM mode.

Chromatographic separation was performed on an Acquity UPLC BEH C18 column (2.1 × 100 mm, 1.7 µm) at 40°C, with a flow rate of 0.3 mL/min using a binary gradient. The injection volume was 10 µL. Mobile phase A consisted of 5 mM ammonium formate (Aladdin Scientific) in water:acetonitrile (35:65), and mobile phase B was 5 mM ammonium formate in acetonitrile:water (65:35). The gradient started at 65% B for column equilibration, followed by gradient elution. Mass spectrometry was performed in negative ion mode using an electrospray ionization (ESI) source on an Agilent 6410 QQQ LC‐MS/MS system. Data were acquired and analyzed with MassHunter Workstation (v06.00, Agilent Technologies).

### 4.13 Untargeted Lipidomics Analysis

Pre‐treated cells were collected for lipid extraction. Briefly, 200 µL of water was added to the sample, vortexed for 5 s, followed by 240 µL precooled methanol and vortexed for 30 s. Then, 800 µL Methyl tert‐butyl ether (MTBE) (Thermo Fisher Scientific) was added, sonicated for 20 min at 4°C, and centrifuged at 14000 × g for 15 min at 10°C. The upper organic phase was collected and dried under nitrogen for 2 hours. LC separation was performed using a CSH C18 column (1.7 µm, 2.1 mm × 100 mm). 3 µL of lipid extract was injected. Solvent A consisted of acetonitrile–water (6:4, v/v) with 0.1% formic acid and 0.1 mM ammonium formate; solvent B was acetonitrile–isopropanol (1:9, v/v) with the same additives. The initial mobile phase was 30% solvent B at 300 µL/min for 2 min, increased to 100% solvent B over 23 min, and equilibrated at 5% solvent B for 10 min. Mass spectra were acquired on a Q‐Exactive (Thermo Fisher Scientific) Plus in positive and negative modes. ESI parameters were: source temperature 300°C, capillary temperature 350°C, ion spray voltage 3000V, S‐Lens RF level 50%, and scan range m/z 200–1800.

### 4.14 RNA Sequencing (RNA‐Seq) Analysis

Briefly, total RNA was isolated using the RNA extraction kit (ES Science) according to the manufacturer's protocol. Library preparation and sequencing were performed by Applied Protein Technology Co., Ltd., using 400 ng of input RNA per sample. The sequencing libraries were then constructed and run on the Illumina NovaSeq 6000 platform. Differential gene expression was analyzed using the Wald statistical test, with genes considered significantly altered at *P* < 0.05.

### 4.15 Immunohistochemistry (IHC)

IHC was performed as previously described [24]. Briefly, antigen retrieval was done by heating slides in a cooker at 125°C for 30 seconds. Paraffin‐embedded tissue was incubated overnight at 4°C with the primary antibody, followed by secondary antibody application and detection with DAB substrate. Immunostaining was independently reviewed and scored by two pathologists. The Reactive Score (RS) was calculated by multiplying the percentage of positive cells (0‐100%) by the staining intensity (0‐4 scale) and dividing by 10. Intensity was scored as: 0 (no staining), 1 (weak), 2 (moderate), 3 (strong), and 4 (very strong).

### 4.16 Molecular Docking and Virtual Screening

The structures of PHB2 and ACSL3 proteins were predicted using AlphaFold3, followed by molecular dynamics simulations and clustering of structural trajectories. Virtual screening was performed in the region between the two proteins, with the screening pocket on ACSL3 (indicated by the black rectangular area in Figure S4B–D). The protein structure was optimized using Schrödinger's Protein Preparation Wizard, which included bond order optimization, hydrogen addition, disulfide bond assignment, and protonation at pH 7.0 using the PROPKA method. Energy minimization was carried out with the OPLS4 force field, eliminating atomic clashes and achieving heavy atom RMSD convergence to 0.3 Å.

Virtual screening was performed using the TargetMol compound library (T001). The library was processed with Schrödinger's LigPrep module, where compounds were protonated at pH 7.0±2.0 using the Epik method, salts were removed, tautomers generated, and atomic chirality preserved. Conformers (up to 32 per compound) were generated to ensure correct global conformations. The prepared library was then used as the ligand file for screening with Glide SP (Standard Precision mode)‐MMGBSA. Flexible docking with energy optimization was applied using default parameters. The top 10% of conformers were evaluated for binding free energy with MMGBSA. Redundant enantiomers and tautomers were removed, retaining the best‐scored conformers. Conformers with an SP mode docking score below ‐6 kcal/mol, ligand strain energy under 10 kcal/mol, and binding free energy below −50 kcal/mol were selected for protein‐ligand interaction fingerprint (PLIF) analysis, clustering analysis, and binding mode analysis.

### 4.17 Surface Plasmon Resonance (SPR) Assay

Affinity and kinetic interactions were assessed using SPR on the Biacore T200 system (Cytiva). Recombinant ACSL3 proteins were immobilized on separate lanes of a CM5 sensor chip by coupling with 10 mm acetate buffer (pH 4.5) via standard EDC‐mediated amine coupling, according to the manufacturer's protocol (Cytiva). Single‐domain antibodies were analyzed at 25°C by injecting five concentrations (0‐50 µM) at a flow rate of 30 µL/min for 90 seconds, followed by a 200‐second dissociation phase. Data were processed using Biacore T200 Evaluation software.

### 4.18 ssGSEA Analysis

ssGSEA was performed using the GSVA (v1.30.0) R package (method = “ssgsea”) on PHB2 gene expression data from the TCGA Stomach Adenocarcinoma (STAD) dataset. The ‘GSEA_WP_FERROPTOSIS’ gene signature was used to calculate ssGSEA scores.

### 4.19 Cell Line‐Derived Xenograft Model

Briefly, 5‐week‐old female BALB/c nude mice (purchased from Gempharmatech, Nanjing, China) were subcutaneously injected with the indicated cells (5 × 10^^6^). Tumor growth was monitored every three days by measuring tumor length and width with calipers, and volumes were calculated using the formula: volume (mm^3^) = ½ × length × width^2^. After 21 days of treatment, the mice were euthanized, and tumors were excised and weighed. Tumor tissues were fixed in formalin, embedded in paraffin, and subjected to hematoxylin and eosin (H&E) staining. IHC analysis was subsequently performed to assess Ki67 and 4‐HNE expression. All animal experiments were approved by the Animal Ethics Committee of Anhui Medical University (approval number: LLSC20250591).

### 4.20 Patient‐Derived Organoid (PDO) Model

GC PDOs were generated from fresh tumor tissues obtained during surgical resection from patients who had previously received CDDP‐containing chemotherapy but exhibited relative resistance, as reflected by less than 50% growth inhibition, with informed patient consent and institutional ethical approval (Anhui Medical University, Approval No. 81250111). Tumor samples were minced and sequentially digested with MasterAim Tissue Digestion Solutions I and II at 37°C to obtain small cell clusters (<200 µm). After filtration and red blood cell removal, cells were counted, resuspended in Matrigel (4000–6000 cells/µL), and plated as 50 µL droplets in 24‐well plates. After Matrigel solidification, MasterAim complete organoid culture medium was added. Organoids were cultured at 37°C in 5% CO_2_, with medium changes every 3–4 days, and passaged every 10–15 days.

### 4.21 Serum Biochemical Analysis

Mouse serum was collected at the experimental endpoint to evaluate systemic toxicity. Serum ALT (Cat. No. C009‐2‐1), AST (Cat. No. C010‐2‐1), BUN (Cat. No. C013‐1‐1), and Cr (Cat. No. C011‐2‐1) were measured using commercial kits from Nanjing Jiancheng Bioengineering Institute according to the manufacturer's protocols. ALT and AST were quantified by Reitman–Frankel colorimetric assays at 505 nm, BUN by the diacetyl monoxime method at 520 nm after heating at 95–100°C for 15 min, and Cr by a sarcosine oxidase‐based enzymatic assay at 546 nm. The resulting biochemical parameters were used to assess hepatic and renal function in mice.

### 4.22 Statistics and Reproducibility

All experimental data are presented as mean ± standard. Statistical analyses were conducted using GraphPad Prism version 10.1.2. Comparisons between two groups were made using unpaired, two‐tailed Student's t‐tests, while one‐way or two‐way ANOVA followed by Tukey's post hoc test was used for comparisons among multiple groups. *P* value less than 0.05 was considered statistically significant.

Sample sizes were not predetermined by statistical methods but were based on prior experimental experience and relevant literature. Sample sizes and replicates are detailed in the corresponding figure legends. No data points were excluded from analysis.

## Author Contributions

**Liang Xu**: conceptualization, methodology, validation, formal analysis, investigation, data curation, writing – original draft preparation. **Wanying Xiang**: methodology, validation, investigation. **Xinyue Wang**: formal analysis. **Yue Pan**: investigation. **Jing Gao**: validation, investigation. **Jiezhen Yang**: formal analysis, investigation. **Yufei Wang**: investigation. **Zhipu Zhu**: investigation. **Man Tong**: data curation, writing – review and editing. **Lei Jin**: data curation, writing – original draft preparation, writing – review and editing, supervision. **Yan Ye**: conceptualization, data curation, writing – original draft preparation, writing – review and editing, supervision. All authors have read and agreed to the published version of the manuscript.

## Conflicts of Interest

The authors declare no conflicts of interest.

## Supporting information




**Supporting File**: advs77350‐sup‐0001‐SuppMat.pdf.

## Data Availability

The data that supports the findings of this study are available in the supplementary material of this article.
