# Supplementary material for: Targeting the PHB2–ACSL3 Lipid‐Remodeling Axis Overcomes Cisplatin Resistance by Restoring Ferroptosis in Gastric Cancer
Source: Adv Sci (Weinh). 2026 Aug 21;13(63):e77350. doi: 10.1002/advs.77350 (PMC13496284; doi:10.1002/advs.77350)
Supplement: Supplementary file 1 [file ADVS-13-e77350-s001.pdf]

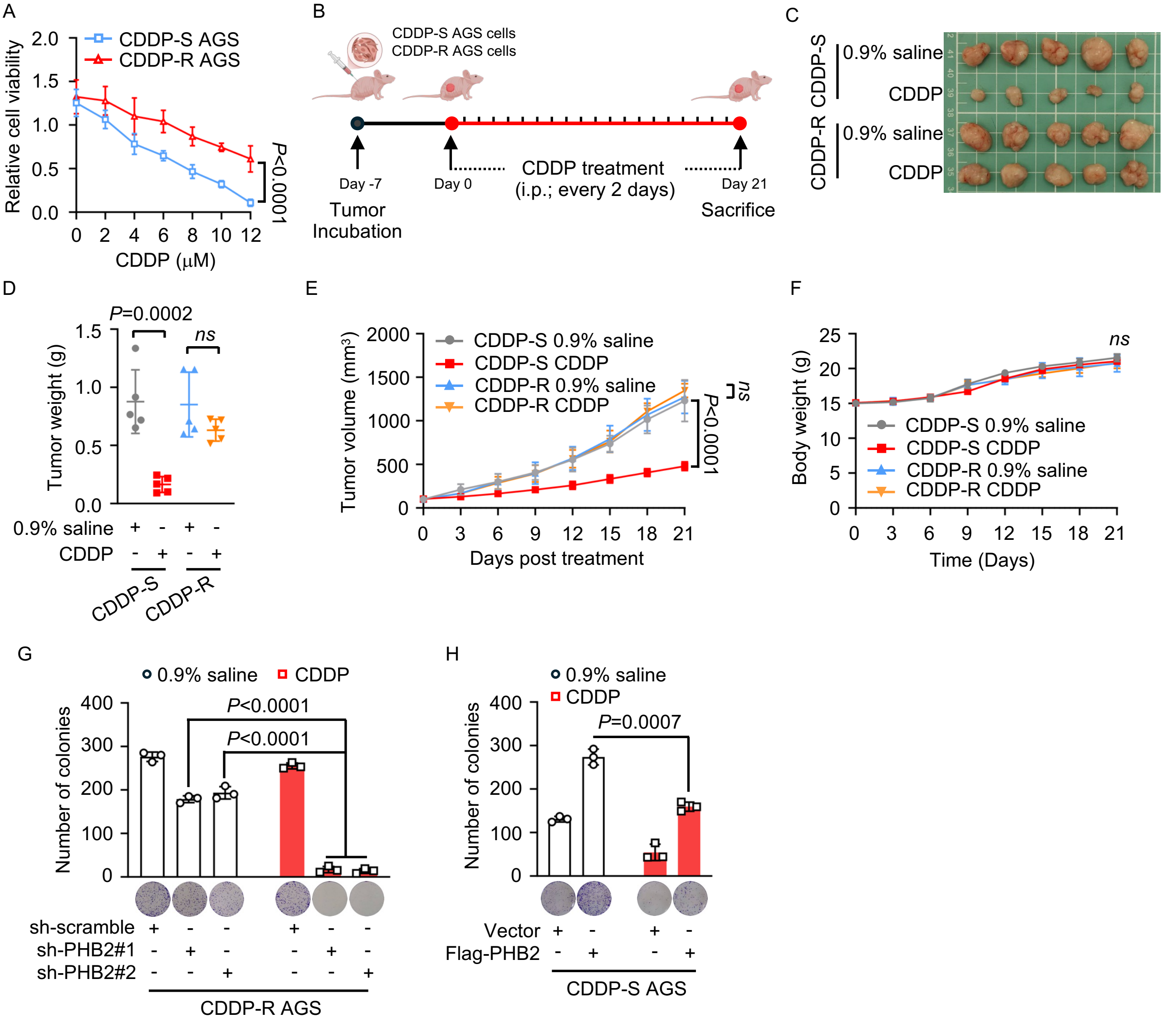

Supplementary Figure 1. Establishment of CDDP-R and CDDP-S AGS xenograft tumors. A CDDP-R and CDDP-S AGS cells were treated with the indicated concentrations of CDDP for 72 hours, and cell viability was assessed using the MTT assay. **B** Schematic illustration of the establishment of CDDP-R and CDDP-S AGS xenograft tumors. BALB/c nude mice bearing AGS-derived xenografts were treated with either 0.9% saline or cisplatin (5 mg/kg, intraperitoneally [i.p.]) every 2 days for 3 weeks (n = 5 per group). **C-E** Representative tumor images (C), tumor weights (D), and growth curves (E) of xenograft tumors. Scale bar, 1 cm. **F** Body weights of mice in each group were monitored every 3 days for 3 weeks. **G, H** PHB2 knockdown sensitized CDDP-R AGS cells to CDDP (G), whereas PHB2 overexpression conferred resistance (H), as demonstrated by clonogenic assays.

Data represent three independent experiments and are presented as mean ± SD. one-way (D, G, H) or two-way ANOVA followed by Tukey's multiple comparison (A, E, F).

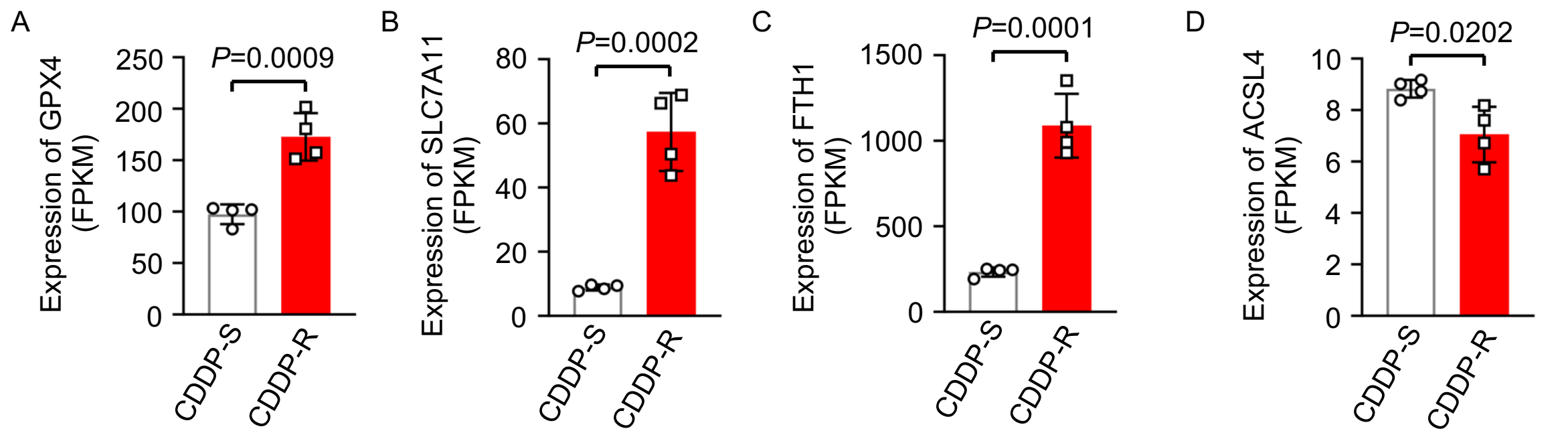

Supplementary Figure 2. Ferroptosis-related gene expression in CDDP-R vs. sensitive AGS tumors. A-D The mRNA expression levels of ferroptosis-related genes GPX4 (A), SLC7A11 (B), FTH1 (C), and ACSL4 (D) from RNA-seq analysis of CDDP-R and CDDP-S AGS xenograft tumors.

Data are presented as mean ± SD. Two-tailed Student's t-test (A-D).

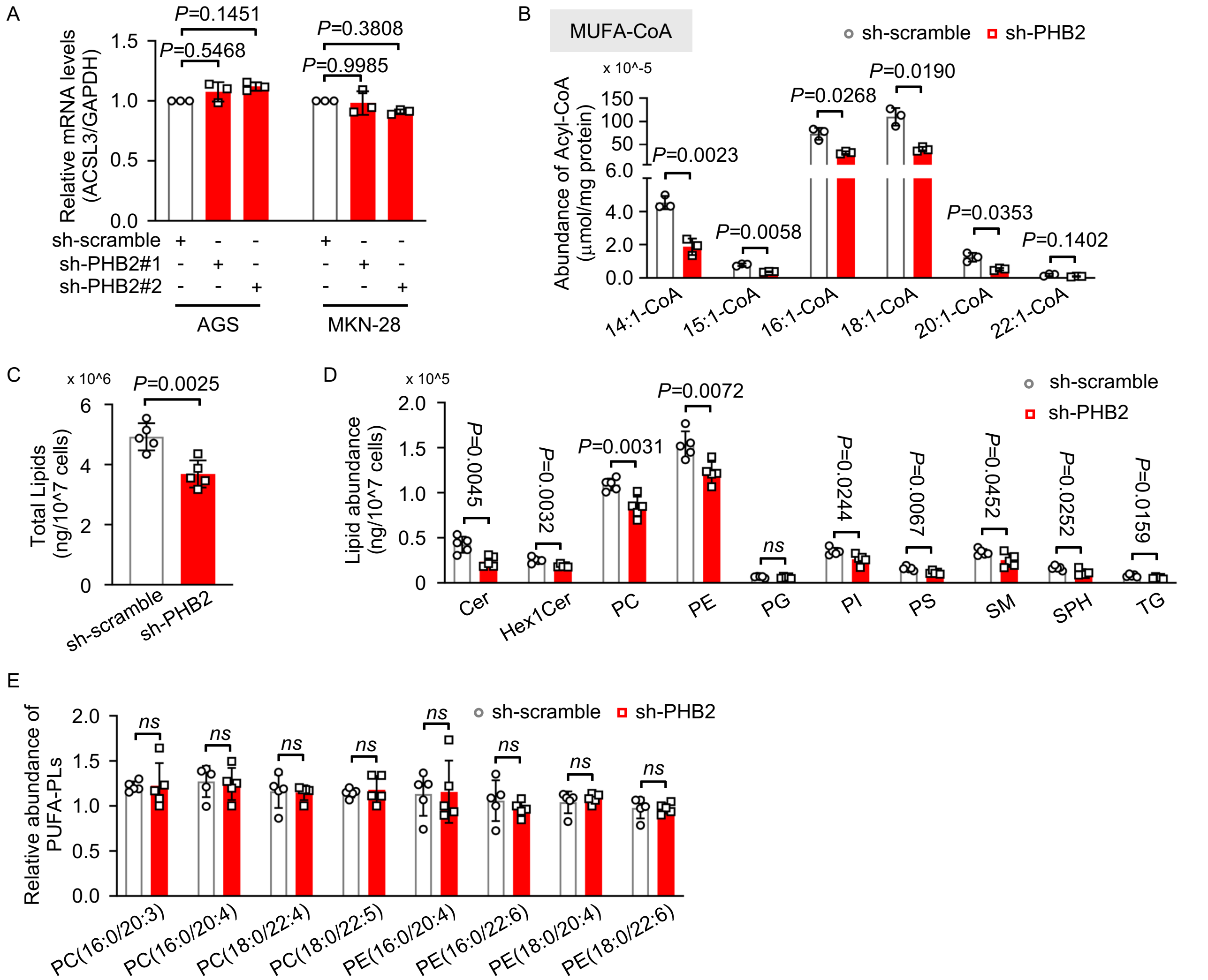

Supplementary Figure 3. PHB2 knockdown alters membrane phospholipid composition in GC cells. A qRT-PCR analysis of ACSL3 mRNA levels in AGS.sh-PHB2 and MKN-28.sh-PHB2 cells. **B** Targeted acyl-CoA profiling demonstrated that PHB2 knockdown reduced levels of several MUFA-CoA species, including 14:1-CoA, 15:1-CoA, 16:1-CoA, 18:1-CoA and 20:1-CoA. **C, D** Untargeted lipidomics profile revealed that PHB2 knockdown led to a reduction in total lipid content (C) and a decrease in most lipid species, including Cer, Hex1Cer, PC, PE, PI, PS, SM, SPH, and TG, although the change in PG was not statistically significant (D). Cer, Ceramide; Hex1Cer, Monohexosylceramide; PG, Phosphatidylglycerol; PI, Phosphatidylinositol; PS, Phosphatidylserine; SM, Sphingomyelin; SPH, Sphingosine; TG, Triglycerides. **E** LC-PUFA-containing PLs remained unchanged upon PHB2 silencing in AGS cells.

Data are presented as mean ± SD. Two-tailed Student's t test (B-E). One-way ANOVA followed by Tukey's multiple comparison (A).

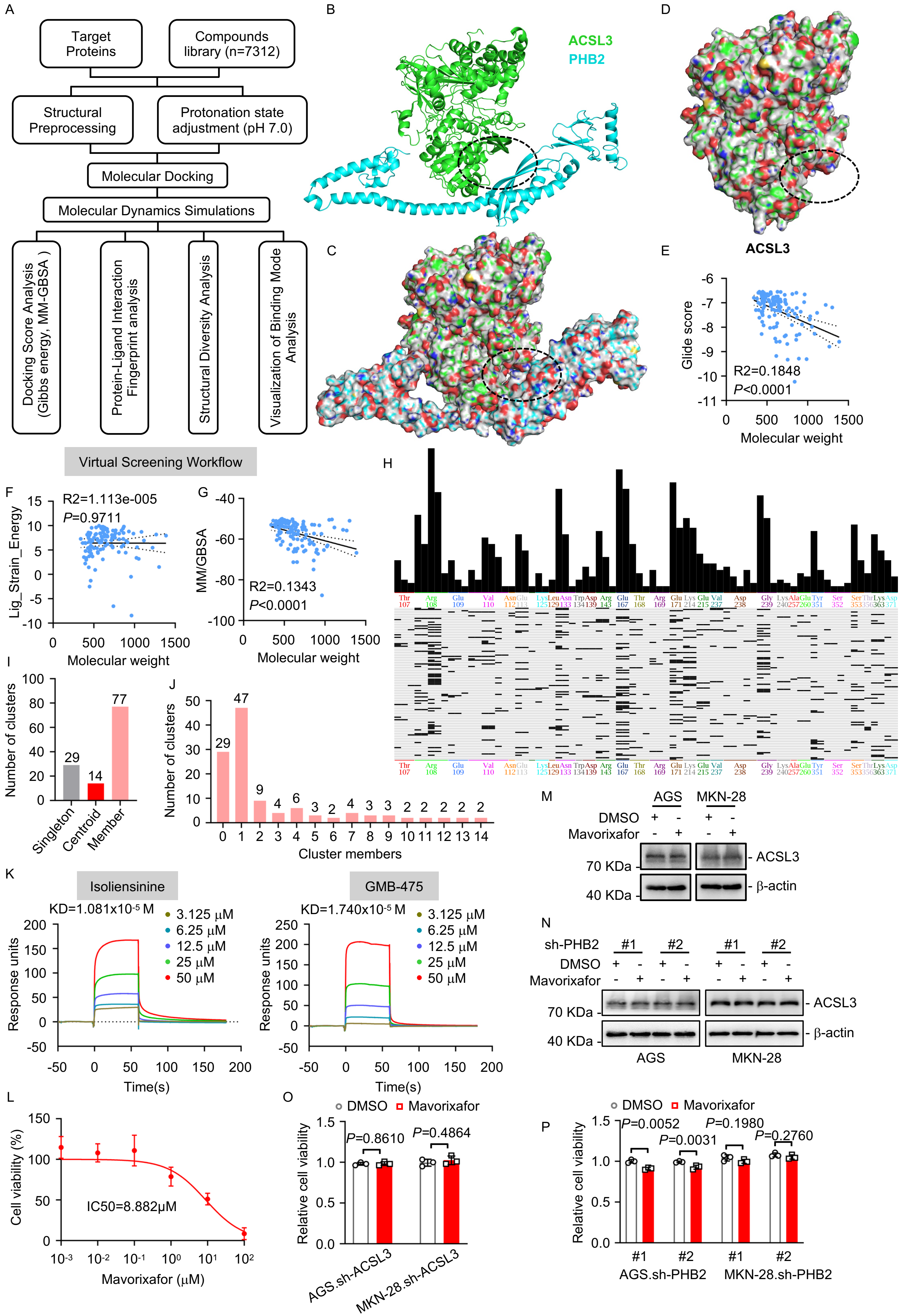
A
Target Proteins
Compounds library (n=7312)
Structural Preprocessing
Protonation state adjustment (pH 7.0)
Molecular Docking
Molecular Dynamics Simulations
Docking Score Analysis (Gibbs energy, MM-GBSA)
Protein-Ligand Interaction Fingerprint analysis
Structural Diversity Analysis
Visualization of Binding Mode Analysis
Virtual Screening Workflow
B
ACSL3
PHB2
C
D
ACSL3
E
Glide score
R2=0.1848
P<0.0001
Molecular weight
F
Lig_Strain_Energy
R2=1.113e-005
P=0.9711
Molecular weight
G
MM/GBSA
R2=0.1343
P<0.0001
Molecular weight
H
I
Number of clusters
29
14
77
Singleton
Centroid
Member
J
Number of clusters
29 47 9 4 6 3 2 4 3 3 2 2 2 2 2
Cluster members
K
Isoliensinine
KD=1.081x10-5 M
GMB-475
KD=1.740x10-5 M
3.125 μM
6.25 μM
12.5 μM
25 μM
50 μM
Response units
Time(s)
L
Cell viability (%)
IC50=8.882μM
Mavorixafor (μM)
M
AGS
MKN-28
DMSO
Mavorixafor
70 KDa
40 KDa
ACSL3
β-actin
N
sh-PHB2
#1
#2
DMSO
Mavorixafor
70 KDa
40 KDa
ACSL3
β-actin
AGS
MKN-28
O
DMSO
Mavorixafor
Relative cell viability
P=0.8610
P=0.4864
AGS.sh-ACSL3
MKN-28.sh-ACSL3
P
DMSO
Mavorixafor
Relative cell viability
P=0.0052
P=0.0031
P=0.1980
P=0.2760
#1
#2
AGS.sh-PHB2
MKN-28.sh-PHB2

Supplementary Figure 4. Molecular docking and virtual screening. A The workflow diagram of the virtual screening process. **B-D** The structures of PHB2 and ACSL3 proteins were predicted using AlphaFold3, and virtual screening was conducted in the region between the two proteins, with the screening pocket on ACSL3 highlighted by the black rectangular area. **E-G** The molecular weight-score distribution includes molecular weight versus docking score (E), ligand strain energy (F), and binding free energy (G). **H** Protein Ligand Interaction Fingerprints (PLIF) analysis of 120 screened compounds, with identical colors representing the same amino acids. Higher vertical density indicates a greater frequency of interactions between the amino acids and the compounds. **I, J** Clustering analysis of the 120 screened compound structures: "Singleton" refers to Class 0 molecules with no structural similarity to others, each forming a unique cluster; "Centroid" denotes the central molecule within a cluster; and "Member" indicates the other molecules in the same cluster (I). Non-zero integers represent clusters with structurally similar molecules, while Class 0 includes structurally unique compounds (J). **K** SPR analysis of the binding affinity between immobilized recombinant human ACSL3 and serially diluted concentrations of Isoliensinine (KD=1.081×10^{-5} M; left) and GMB-475 (KD=1.740×10^{-5} M; right). **L** IC_{50} curve of Mavorixafor at 72 hours, with cell viability measured by MTT assay. **M, N** Western blot analysis of ACSL3 protein levels in control (M) and PHB2-knockdown (N) AGS and MKN-28 cells following Mavorixafor treatment. **O, P** MTT analysis of cell viability in ACSL3-knockdown (O) and PHB2-knockdown (P) AGS and MKN-28 cells following Mavorixafor treatment.

Data represent three independent experiments and are presented as mean ± SD. Two-tailed Student's t-test (O, P).

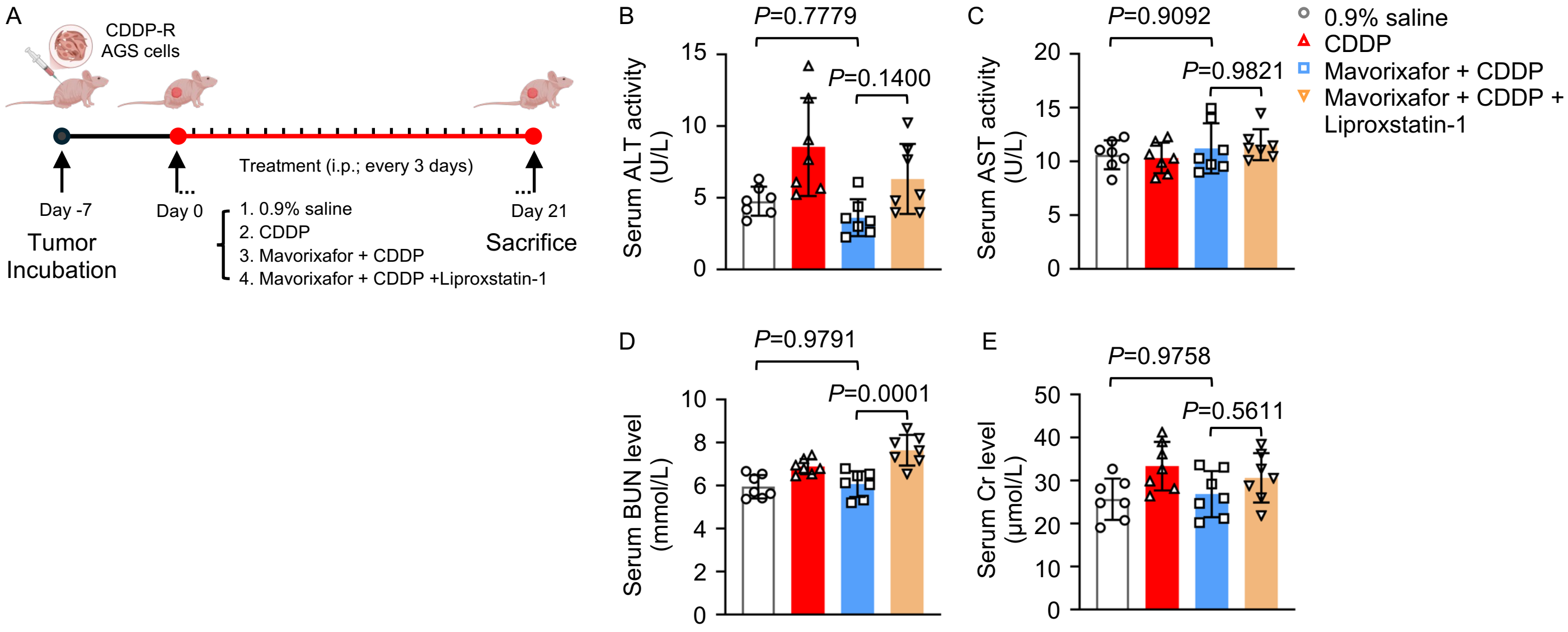

Supplementary Figure 5. Treatment scheme and systemic toxicity assessment in the CDDP-R AGS xenograft model. A Schematic diagram of treatment groups in the CDDP-R AGS xenograft tumors model (n = 7 per group). **B-E** Serum ALT (B), AST (C), BUN (D) and Cr (E) levels were measured by biochemical assays at the experimental endpoint across the indicated treatment groups. Minor variations were observed, but all values remained within the expected physiological range.

Data represent three independent experiments and are presented as mean ± SD. One-way ANOVA test followed by Tukey's multiple comparisons (B-E).

Supplementary Table 1. List of siRNAs and shRNAs

PHB2 shRNA#1	ccgggcTGAGCTTTAGCCGAGAGTActcgagTACTCTCGGCTAAAGCTCAgctttttg
PHB2 shRNA#2	ccggGAGCAAGAACCCTGGCTACATctcgagATGTAGCCAGGGTTCTTGCTCtttttg
ACSL3 shRNA#1	ggatgtgatactttagataaaTTCAAGAGAtttatctaaagtatcacatcc
ACSL3 shRNA#2	ggttattcttggacagtataaTTCAAGAGAttatactgtccaagaataacc
PHB2 siRNA#1	5'- GUGAUUUCCUACAGUGUUGUUCCCUTT-3'
PHB2 siRNA#2	5'- AGAUAAACACCAACCCAGGAAUUCUTT-3'
ACSL3 siRNA#1	5'- GCUGAAGUCUGUUAAUUCUTT-3'
ACSL3 siRNA#2	5'- CCCGAUGGAUGCUUAAAGATT-3'

Supplementary Table 2. Top 50 compounds selected by binding free energy

Formula	ID	Name	MMGBSA
C48H82O19	TQ0174	**Mavorixafor**	-87.64
C37H42N2O6	T5S1103	Isoliensinine	-71.35
C43H46F3N7O7S	T8488	GMB-475	-70.49
C45H52ClN9O8S	T16153	MS4078	-67.21
C33H40O19	T5S1526	Apigenin-7-O-(2G-rhamnosyl)gentiobioside	-66.06
C25H43NO18	T0247	Acarbose	-66
C29H36O16	T5796	Plantainoside D	-65.92
C39H40N10O7	T36694	XY028-140	-65.55
C41H61F3N12O13	T7616L	A 779 3TFA(159432-28-7(free base))	-65.37
C47H66N10O8	T12506L1	PMX 205 acetate(514814-49-4 free base)	-65.14
C37H48N4O5	T1623	Lopinavir	-64.87
C30H33N3O5S	T63872	KU-60019	-64.87
C32H38O15	TN1611	Epimedoside A	-64.63
C34H36F3NO13	T7604	Valrubicin	-63.43
C21H42N6O5	T7676	LSKL, Inhibitor of Thrombospondin TSP-1	-62.83
C28H32N8O6	T7683	Adenosine amine congener	-62.73
C25H48N14O16S2	T1441	Capreomycin sulfate	-62.67
C41H43ClFN9O6	T22263	Bavdegalutamide	-62.28
C21H17Cl3N4OS	T3045	Salubrinal	-61.16
C22H26N6O2	T16429	GNE 2861	-60.47
C26H29N5O2	T30855	CGP77675	-59.67
C24H32ClNO3	T5380	Ansofaxine hydrochloride	-58.07
C29H53N3O12S	T17594	Biotin-PEG8-acid	-57.41
C22H28ClNaO6	T5847	Cloprostenol sodium salt	-57.17
C29H31NO4	T12654	(Rac)-Acolbifene	-56.66
C33H31N3O7	T9616	PD-1/PD-L1-IN-10	-55.58
C30H32O15	TN1067	Galloylpaeoniflorin	-55.11
C20H18O11	T7781	Reynoutrin	-54.79
C17H19Cl2N5O2S	T11319	FR183998 free base	-54.32
C34H37N3O6	TN6088	tricoumaroyl spermidine	-54.25
C29H24O12	T7602	Theaflavin	-54.13
C31H36F3N7O5	T10539	BIBO3304 TFA	-54.08
C24H27NO4	T22553	Adaphostin	-54.01
C26H22N2O2	T3953	Salermide	-53.74
C25H20N6O2S	T30544	BMS-919373	-53.67
C22H29NO2	T12758	Ro 25-6981	-52.96
C25H23ClN6	T6158	LDN-193189 HCl	-52.72
C28H30N4O6S	T9396	FMoc-Arg(Tos)-OH	-52.38
C25H24O12	T5736	1,3-Dicaffeoylquinic acid	-52.23
C24H19BFNO5S	T11533	HA155	-52.18
C19H23ClN4O7	T18818	Thalidomide-O-amido-PEG-C2-NH2 hydrochloride	-52.14
C23H34N5O14P	T2409L	Tenofovir Disoproxil Fumarate	-52.05
C24H40N2O12	T15960	Mal-amido-PEG7-acid	-51.78
C25H40ClN3O8	T5028	Landiolol hydrochloride	-51.72
C25H28N6O2	T3072	XL019	-51.71
C30H18O10	T3417	Amentoflavone	-50.96
C20H37N3O13	T1022	Hygromycin B	-50.96
C22H21Cl2N5O	T3182	L-778123 hydrochloride	-50.93
C24H32N4O6	T9458	PF-00835231	-50.04
